# Integrative genetic, epigenetic and pathological analysis of paraganglioma reveals complex dysregulation of NOTCH signaling

**DOI:** 10.1007/s00401-013-1165-y

**Published:** 2013-08-18

**Authors:** Alessandro Cama, Fabio Verginelli, Lavinia Vittoria Lotti, Francesco Napolitano, Annalisa Morgano, Andria D’Orazio, Michele Vacca, Silvia Perconti, Felice Pepe, Federico Romani, Francesca Vitullo, Filippo di Lella, Rosa Visone, Massimo Mannelli, Hartmut P. H. Neumann, Giancarlo Raiconi, Carlo Paties, Antonio Moschetta, Roberto Tagliaferri, Angelo Veronese, Mario Sanna, Renato Mariani-Costantini

**Affiliations:** 1 Unit of General Pathology, Aging Research Center (Ce.S.I.), G. d’Annunzio University Foundation, Via Colle dell’Ara, 66100 Chieti, Italy; 2 Department of Pharmacy, G. d’Annunzio University, Via dei Vestini 1, 66100 Chieti, Italy; 3 Department of Experimental Medicine, University La Sapienza, Viale Regina Elena 324, 00161 Rome, Italy; 4 NeuRoNe Lab, Department of Informatics, University of Salerno, Via Ponte Don Melillo, 84084 Fisciano, Salerno Italy; 5 Laboratory of Lipid Metabolism and Cancer, Department of Translational Pharmacology, Consorzio Mario Negri Sud, Via Nazionale 8/A, 66030 Santa Maria Imbaro, Chieti Italy; 6 IRCCS National Cancer Research Center Giovanni Paolo II, Viale Orazio Flacco 65, 70124 Bari, Italy; 7 Gruppo Otologico, Via Emmanueli 42, 29100 Piacenza, Italy; 8 Department of Medical, Oral and Biotechnological Sciences, G. d’Annunzio University, Via dei Vestini 1, 66100 Chieti, Italy; 9 Department of Experimental and Clinical Biomedical Sciences, University of Florence, Viale Morgagni 50, 50134 Florence, Italy; 10 Section of Preventive Medicine, Department of Nephrology, Albert-Ludwigs-University of Freiburg, Hugstetter Strasse 55, 79106 Freiburg, Germany; 11 Unit of Anatomic Pathology, Department of Clinical Pathology, Hospital G. da Saliceto, Via Giuseppe Taverna 49, 29100 Piacenza, Italy

**Keywords:** Paraganglioma, Head and neck, NOTCH signaling, CNV, MicroRNA, Paraganglioma cell culture

## Abstract

Head and neck paragangliomas, rare neoplasms of the paraganglia composed of nests of neurosecretory and glial cells embedded in vascular stroma, provide a remarkable example of organoid tumor architecture. To identify genes and pathways commonly deregulated in head and neck paraganglioma, we integrated high-density genome-wide copy number variation (CNV) analysis with microRNA and immunomorphological studies. Gene-centric CNV analysis of 24 cases identified a list of 104 genes most significantly targeted by tumor-associated alterations. The “NOTCH signaling pathway” was the most significantly enriched term in the list (*P* = 0.002 after Bonferroni or Benjamini correction). Expression of the relevant NOTCH pathway proteins in sustentacular (glial), chief (neuroendocrine) and endothelial cells was confirmed by immunohistochemistry in 47 head and neck paraganglioma cases. There were no relationships between level and pattern of NOTCH1/JAG2 protein expression and germline mutation status in the *SDH* genes, implicated in paraganglioma predisposition, or the presence/absence of immunostaining for SDHB, a surrogate marker of *SDH* mutations. Interestingly, NOTCH upregulation was observed also in cases with no evidence of CNVs at NOTCH signaling genes, suggesting altered epigenetic modulation of this pathway. To address this issue we performed microarray-based microRNA expression analyses. Notably 5 microRNAs (miR-200a,b,c and miR-34b,c), including those most downregulated in the tumors, correlated to NOTCH signaling and directly targeted *NOTCH1* in in vitro experiments using SH-SY5Y neuroblastoma cells. Furthermore, lentiviral transduction of miR-200s and miR-34s in patient-derived primary tympano-jugular paraganglioma cell cultures was associated with *NOTCH1* downregulation and increased levels of markers of cell toxicity and cell death. Taken together, our results provide an integrated view of common molecular alterations associated with head and neck paraganglioma and reveal an essential role of NOTCH pathway deregulation in this tumor type.

**Electronic supplementary material:**

The online version of this article (doi:10.1007/s00401-013-1165-y) contains supplementary material, which is available to authorized users.

## Introduction

Paragangliomas (PGLs), rare, weakly metastatic but invasive neoplasms of the paraganglia, provide an example of organoid tumorigenesis from neural crest-derived cells belonging to the autonomic nervous system. As paraganglia, PGLs can be catecholamine-secreting (chromaffin), mostly thoraco-abdominal (including pheochromocytomas), or non-chromaffin, mostly in the head and neck [15, 32]. Head and neck PGLs account for about 0.6 % of all head and neck tumors, usually present between the 4th and 6th decades of life, and mostly arise from paraganglia at the carotid bifurcation, in or around the jugular bulb, in the cervical tract of the vagus, or within the temporal bone. These PGLs cause important morbidity and are potentially lethal, due to the anatomic region of onset [32].

At least one-third of all PGLs have a hereditary basis, often blurred by incomplete penetrance or imprinting [18]. The susceptibility genes include *SDHA*, *SDHB*, *SDHC*, *SDHD*, and *SDHAF2*, encoding mitochondrial complex II components; and, with lower frequencies, *VHL* and *PHD2* (*EGLN1*), that regulate HIFα; the MYC regulator *MAX*; *RET*, implicated in glial neurotrophic signaling; *NF1*, which controls glial tumorigenesis; *TMEM127*, associated with mTOR signaling, and *KIF1B*β, involved in mitochondrial transport and apoptosis [6, 8, 18, 25, 39, 47].

This genetic heterogeneity contrasts with the substantially monotonous tumor phenotype, which mimics paragangliar histoarchitecture. In fact, PGLs are organized in interconnected cell clusters (“zellballen”), composed of neurosecretory (chief) cells encircled by glial (sustentacular) cells, embedded in angiomatous stroma [32]. At the somatic level, the molecular pathways involved in PGL are poorly defined [9, 18].

Our goals were to identify candidate molecular pathway(s) commonly affected by genomic alterations in head and neck PGLs, characterize the expression patterns of the pertinent gene products and assess the possible involvement of microRNAs in their deregulation.

## Patients and methods

### Cases and controls

The study was approved by the Bioethical Committee of *G.* *d’Annunzio* University. Blood and tumor samples were from consenting consecutive patients operated at the *Gruppo* *Otologico* clinic, Piacenza, Italy. Only one patient reported PGL family history, only one was positive for metastases (regional lymph nodes). Samples for nucleic acid analyses were stored at −80 °C in RNALater (Qiagen). Fresh samples were also obtained for immunofluorescence (IF), electron microscopy (EM) and cryoimmuno-electron microscopy (cryo-IEM). Overall, 28 cases with 29 tumors (one patient was affected with two synchronous PGLs) yielded nucleic acids adequate for the study (Supplementary Table 1, Online Resource 1). Based on quality/quantity of nucleic acids, 23 cases, with 24 independent tumors, were selected for CNV analysis, 14 samples from 13 independent tumors were used for miRNA expression profiling and 16 samples from 15 independent tumors for quantitative reverse-transcriptase real-time (qRT-PCR) (two distinct samples from tumor 33PT were analyzed in these assays). One tumor (case PTJ64) was used to establish primary cultures of PGL cells. Formalin-fixed, paraffin-embedded (FFPE) tissue blocks qualitatively and quantitatively adequate for standard immunohistochemistry (IHC) could be retrieved for 22 prospectively collected tumors (cases 1PTJ through 64PTJ, Supplementary Tables 1–3, Online Resource 1). In addition, 25 archival tumors (from 24 cases, acronyms/R1 through/R24, Supplementary Tables 2 and 3, Online Resource 1) were studied by IHC only. Most cases were tympanic or tympano-jugular PGLs (PTs and PTJs, Supplementary Tables 1 and 2, Online Resource 1), that arise from Jacobson’s nerve (JN), the tympanic branch of the glossopharyngeal nerve (IX cn), from Arnold’s nerve, the auricular branch of the vagus (X cn), or from paraganglia of the jugular bulb [32]. JN, removed in the modified trans-labyrinthine approach for vestibular schwannoma [43], is the only normal tissue histogenetically relevant for head and neck PGL procurable at surgery. Thus, millimeter-sized samples of morphologically normal JN from 18 donors were used as controls for RNA and miRNA expression studies (Supplementary Fig. 1, Online Resource 2; Supplementary Table 4, Online Resource 1).

### Mutational analysis of the *SDH* genes

Germline mutational status (point mutations and large deletions/rearrangements) of the three SDH complex genes strongly associated with PGL development (*SDHB*, *SDHC*, *SDHD*) was assessed according to published procedures [42, 47] on blood samples of 34 cases (9 analyzed at the University of Florence, 25 at Albert-Ludwigs-University, Freiburg). Mutations in *SDHAF2*, which may contribute to PGL in patients negative for mutations in *SDHB*, *SDHC* and *SDHD* [21], were investigated only in the 9 cases analyzed at Florence. Furthermore, a large germline deletion/rearrangement in *SDHB* was detected by CNV analysis and validated by orthogonal assays (as detailed below).

### CNV and gene-centric analyses

Genomic DNA (gDNA) was extracted using DNeasy Blood and Tissue kit (Qiagen), checked by agarose electrophoresis, and quantitated by Qubit fluorometer (Life Technologies). Paired gDNAs (200 ng) from blood and tumor were processed according to the Infinium assay protocol and hybridized on HumanOmni1-Quad BeadChips^®^ (>1 million markers, Illumina), for 16 h. BeadChips were scanned with Illumina Iscan™ and image intensities were extracted and genotyped using Illumina’s Genome Studio 2011.1^®^ software. The SNP genotyping call rate was >99.0 %, indicating high-quality data. Only autosomal SNPs were considered [67]. The data were analyzed with an original framework for computational pipelines management designated *Leaf* [41], that integrates CNV-calling softwares (i.e., PennCNV) with custom CNV data mining procedures to select the CNVs intersecting coding regions, as described in Napolitano et al. [40]. *Leaf* produced a list of the genes most significantly over-represented among those targeted by CNVs (*P* < 0.01 by Fisher’s exact test). This list was submitted to the Database for Annotation, Visualization, and Integrated Discovery (DAVID), which uses fuzzy clustering to group genes into functionally related, statistically ranked classes, based on the similarity of the annotations (http://david.abcc.ncifcrf.gov) [24].

### Orthogonal validation of the CNV hits

Commercial real-time qPCR assays (Life Technologies) were used to validate the CNVs in the *JAG2*, *HES5*, *CTBP1*, *AKIRIN1*, *IDUA* and *PHACTR4* genes. Each qPCR contained the FAM-labeled TaqMan probe for the gene of interest and the VIC-labeled TaqMan probe for the RNaseP reference (4403328, Life Technologies). Each qPCR plate included three no template controls. The relative gene copy numbers were calculated according to manufacturer’s instructions. The C_t_ values were normalized versus the reference C_t_ ($\Delta$C_t_) and the $\Delta\Delta$C_t_ method was computed using the mean of the normal samples as calibrator. The CNVs targeting *NOTCH1*, *DVL1* and *SDHB* were validated by non-fluorescent multiplex-PCR coupled to high-performance liquid chromatography (NFMP-HPLC) [14], using primer pairs in the exonic regions of *NOTCH1* (FW: 5′-AGACGGCATCAACACGGCCTTC-3′, RW: 5′-GTGTAGCTGTCCACGCAGTCCG-3′, 135 bp), *DVL1* (FW: 5′-CCAGACTCATCCGGAAGCACAAACG-3′, RW: 5′-GACGATGTTGAGGGACATGGTGGAG-3′, 206 bp) and SDHB (FW: 5′-CCCGAGGAGCCCAGACAGC-3′, RW: 5′-CCAGCCTTGTCTGGGTCCCATC-3′, 82 bp), together with a set of primers (FW: 5′-TCAGGCTTAGGGTAGAGGACAATG-3′, RW: 5′-TCTGCTTGTAGGGCAACTCG-3′, 94 bp) targeting *PCBD2*, chosen as reference gene because it showed no CNVs in our dataset and in a previous study conducted by quantitative multiplex PCR of short fluorescent fragments [29]. The amplifications were obtained with 24 cycles using a touch-down PCR protocol (denaturation: 15 s at 95 °C; annealing: 15 s at 66 °C with 0.5 C° decrease per cycle; extension: 30 s at 72 °C). The NFMP products were analyzed on a semi-automated DHPLC (Wave 1100, Transgenomic Inc, Omaha, NE) under non-denaturing conditions. The peak heights and ratios were obtained as described [14]. At least two independent experiments, each with triplicate determinations, were performed to validate the selected CNV hits.

### Immunohistochemistry

The PGL cases were chosen for immunohistochemistry (IHC) after revision of all the standard FFPE blocks and hematoxylin–eosin-stained sections for sample quality and quantity. Overall, 47 FFPE tumors from 46 cases were rated as adequate for IHC. These included 22 of the 28 tumors that had been prospectively sampled and for which status at the relevant NOTCH-related genes was assessed by CNV and/or qPCR, and 25 retrospective tumors, for which status at the NOTCH-related genes was unknown (Supplementary Table 3, Online Resource 1). Immunostaining for NOTCH1 (C-20, that recognizes both full-length NOTCH1 and its cleaved intracellular form, Santa Cruz Biotechnology) and JAG2 (Abnova), both diluted 1/50, was performed after heat-induced antigen retrieval (100 °C in Tris–EDTA, pH 9 for 30 min). To characterize the cellular components of the tumors, step sections were incubated with antibodies against: the neuroendocrine marker synaptophysin, strongly expressed in both sustentacular and chief cells (27G121, Novocastra, diluted 1/200, antigen retrieval at 100 °C in citrate buffer, pH 6 for 30 min) [32]; the neurosecretory granule protein chromogranin A, highly expressed in chief cells (5H7, Novocastra; diluted 1/200, antigen retrieval at 100 °C in citrate buffer, pH 6 for 30 min) [32]; the Ca(2+)-binding protein S100, highly expressed in glial tumors (NCL-L-S100p, Novocastra, diluted 1/200, antigen retrieval by trypsin treatment for 30 min) [32]; the mesenchymal intermediate filament vimentin, expressed in immature glia and in endothelia (V9, Novocastra; diluted 1/300, antigen retrieval at 100 °C in citrate buffer, pH 6 for 30 min) [5, 36]; the major anti-apoptotic mitochondrial protein BCL2 (Bcl2/100/D5, Novocastra, diluted 1/30, antigen retrieval at 100 °C in citrate buffer, pH 6 for 30 min) [65]; and the proliferation marker Ki-67 (MM1, Dako; diluted 1/50, antigen retrieval at 100 °C in Tris–EDTA, pH 9 for 30 min) [32].

SDHB IHC, a surrogate marker for mutations in any of the PGL-associated SDH subunit genes [19, 63], was performed using a commercial mouse monoclonal antibody (ABCAM ab14714, clone 21A11, diluted 1/3,000, antigen retrieval at 100 °C in citrate buffer, pH 6 for 30 min) [19].

For all the study antibodies immunostaining was carried out on 5-μm-thick whole sections with 15 min incubation at room temperature, using a Bond Max Immunohistochemical Stainer^®^ (Leica Microsystems, Wetzlar, Germany). Positive and negative control slides were included for each antibody and in each staining batch. The controls for non-specific staining included blocking with normal secondary serum prior to staining with the primary antibody and substitution of normal serum or immunoglobulin G in place of the primary antibody. The results were evaluated both in terms of percentage of positive cells, counted in four high-magnification fields (400×, each field estimated to contain 250–400 cells), and of intensity, scored on a semiquantitative scale (0 = no staining; 1 = weak but definitely positive staining; 2 = moderate staining; 3 = strong staining). NOTCH1, JAG2, S100 and BCL2 were assessed in the three main PGL cell types (chief cells, sustentacular cells and endothelial cells); synaptophysin in chief and sustentacular cells together, as these cell types were similarly and strongly labeled (endothelia were negative), vimentin in all cell types combined (chief, sustentacular and endothelial cells yielded similar staining). Ki67 was evaluated in terms of percentage of positively stained nuclei (chief and/or sustentacular cells), counted in four high-magnification fields. The other routinely assessed clinicopathological variables included vascular invasion, bone infiltration and atypia.

SDHB immunostaining was ranked positive when showing granular cytoplasmic labeling (a mitochondrial pattern), and negative when weak/diffuse or absent, in the presence of positive internal controls (macrophages/monocytes and/or endothelia) [19, 63]. The IHC results were analyzed by two-tailed Student’s *t* test, or Fisher’s exact test, where appropriate.

### Immunofluorescence

Tumor and/or tissue samples were fixed in 4 % buffered paraformaldehyde (PFA) at 4 °C for 5–10 h, followed by 2 % PFA at 4 °C until processing. Cells grown in CultureSlides plates (BD Biosciences) were fixed in 4 % PFA for 30 min at 4 °C, washed in PBS at 4 °C and processed within 2 days. IF was performed as described [56], using the antibodies against NOTCH1, JAG2, chromogranin A, vimentin and S100 detailed above and in Supplementary Table 3, Online Resource 1, plus antibodies to CTBP1 (BD Biosciences) and to the hematopoietic/endothelial markers CD34 and CD31 (Novocastra). Nuclei were stained with DAPI (Sigma-Aldrich). The primary antibodies were visualized using goat anti-mouse IgG fluorescein isothiocyanate-conjugated (Alexa 488, Life Technologies) or goat anti-rabbit IgG Texas-Red-conjugated (Jackson Immuno Research Laboratories Europe). The sections or cells were analyzed using an Apotome Axio Observer Z1 inverted microscope (Carl Zeiss) equipped with an AxioCam MRm Rev.3. Colocalization of signals was analyzed with Axio Vision software release 4.6.3 (Carl Zeiss).

### Electron microscopy

Samples were fixed in 2 % glutaraldehyde in PBS for 24 h at 4 °C, post-fixed in 1 % OsO4 for 2 h, stained for 1 h in 1 % aqueous uranyl acetate, dehydrated with graded acetones and embedded in Epon-812 (Electron Microscopy Sciences). Semithin sections stained with 1 % methylene blue were used to select suitable areas of ultrastructural sectioning. Uranyl acetate/lead citrate-stained ultrathin sections were examined with a Philips CM10 transmission electron microscope (TEM) (FEI).

### Cryo-immunoelectron microscopy

Samples were fixed in 2 % PFA/0.2 % glutaraldehyde in 0.1 M PBS, pH 7.4, for 24 h at 4 °C, then in 2 % formaldehyde overnight at 4 °C. Tissue blocks were embedded into 10 % gelatin (Sigma-Aldrich) in 0.1 M PBS, pH 7.4, solidified on ice, infused in 2.3 M sucrose overnight at 4 °C, mounted on aluminum pins and frozen in liquid nitrogen. Ultrathin cryosections (60 nm) collected with 1 % methylcellulose in 1.15 M sucrose were immunolabeled with primary antibodies to NOTCH1, S100, chromogranin A, and vimentin, as described [56]. Bound antibodies were visualized using goat anti-mouse conjugated with 15-nm gold (British BioCell International) or protein-A conjugated with 10-nm gold (G. Posthuma, Utrecht, The Netherlands). Cryosections were analyzed with a Philips CM10 TEM.

### MiRNA array

Total RNA was purified using miRNeasy Mini Kit (Qiagen) and qualitatively checked using Experion (Bio-Rad Laboratories) and spectrophotometry. The RNA samples were selected based on relative quality index (RQI >4.5). MiRNA profiling was performed on 14 PGL samples from 13 independent tumors (two distinct areas from tumor 33PT were analyzed in this assay; Supplementary Table 1, Online Resource 1) and 13 Jacobson’s nerves (JNs, Supplementary Table 4, Online Resource 1). Because of the generally minute sample sizes and of the inherently low RNA yields, we were forced to pool the JN RNAs in 5 sets including 2–5 nerves, each contributing the same RNA quantity. The experiments were conducted using the Human v2 MicroRNA Expression Profiling Kit (1146 human miRNAs, >97 % coverage of miRBase v12) and GoldenGate GT Universal BeadChips on an Illumina IScan™ reader. The data were processed through Illumina Genome Studio signal filtration and cleaning algorithms. The miRNAs differentially expressed between the PGL and the JN groups were identified using the differential analysis function of Genome Studio (*P* < 0.05 after FDR adjustment).

### Real-time quantitative reverse transcription PCR (qRT-PCR)

Total RNA from PGLs and JNs was purified using TRIzol (Invitrogen) and quantified with Nanodrop 2000 (Thermo Scientific). The miRNA sequences were from miRBase (http://www.mirbase.org/index.shtml). The stem loop RT primers were designed with a modification to include the Universal Probe Library (UPL) #21 sequence binding site [11, 64]. UPL Probe #21 was from the UPL database (Roche Diagnostics). Oligonucleotides are in Supplementary Table 5 (Online Resource 1). Total RNA (50 ng) was retro-transcribed with the TaqMan Micro-RNA Reverse Transcription Kit (Life Technologies). The reactions were incubated 30 min at 16 °C, followed by pulsed RT of 60 cycles at 30 °C for 30 s, 42 °C for 30 s, and 50 °C for 1 s [59].

The *NOTCH1*, *JAG2*, *HES5* and *HES1* mRNA reference sequences from NCBI were used into the UPL Assay Design Center software (https://www.rocheappliedscience.com/sis/rtpcr/upl/index.jsp?id=uplct_030000) to identify the primers and the UPL probes (Supplementary Table 5, Online Resource 1). Total RNA (700 ng) was retro-transcribed with High Capacity cDNA Reverse Transcription Kit (Life Technologies). The real-time PCRs were performed using an Applied Biosystems 7900 instrument. MiRNA and mRNA levels were measured using Ct (threshold cycle). Target amount, normalized to an endogenous reference (*RNU6* or *ACTB*) and relative to a calibrator, is given by 2^−ΔΔCt^ and/or 2^−ΔCt^ methods.

### Cells

The neuroblastoma cell line SH-SY5Y (ATCC; CRL-2266**)** was acquired in 2007 and authenticated in January 2013 using AmpFlSTR-Identifiler-Plus Kit (Life Technologies). SH-SY5Y cells were cultured in RPMI 1640 (GE Healthcare) with 10 % FBS, 2 mM l-glutamine, 100 IU penicillin, 100 μg/ml streptomycin. HEK293 cells were cultured in DMEM Low Glucose supplemented with 10 % FBS, 2 mM l-glutamine, 100 IU penicillin, 100 μg/ml streptomycin and 50 μg/ml Normocin (Invivogen). Primary PGL cell cultures from a prospectively sampled tympano-jugular PGL case (PTJ64, primary cultures designated PTJ64p) were established following a procedure previously described for primary rat carotid body cultures [44]. In brief, ≈0.5 × 0.5 cm PGL tissue specimens were sampled with sterile equipment in DMEM High Glucose supplied with antibiotics (penicillin, 100 IU; streptomycin, 100 μg/ml; fungizone, 0.25 μg/ml), within 1 min from surgical tumor excision, and maintained at 4 °C during transport to the laboratory (8 h). The samples were enzymatically dissociated as described in Pardal et al. [44]. Cells were cultured in DMEM-F12 (Gibco), supplemented with 20 % FBS and antibiotics as above, at 37 °C, 5 % CO_2_.

### Lentiviral infection

MiRNA-expressing lentiviruses (PMIRH200b-429PA-1, PMIRH34bcPA-1, System Bioscience) were generated using Lentivector-based microRNA precursor constructs (System Biosciences), according to the manufacturer’s instructions. Control lentiviral particles (Cod. SBPMIRH000VA1) were purchased from System Biosciences. PTJ64p cells were seeded at 3.6 × 10^4^ cells per well in 12-well plates in complete culture medium and infected at a multiplicity of infection (MOI) of 50.

### Caspase and toxicity assays

Measurements of caspase activity and of adenylate kinase (AK) release were performed using the Caspase-Glo 3/7^®^ Assay (Promega) and the Toxilight bioassay kit (Lonza, Walkersville, MD), respectively, according to the manufacturer’s protocols, utilizing a VERITAS microplate luminometer (Turner BioSystems). All values were in triplicate and normalized to the controls, both untreated and infected with lentiviral control particles (Cod. SBPMIRH000VA1, System Biosciences).

### Transfection and luciferase assays

MiRNA mimics precursor and negative control were from Life Technologies (Supplementary Table 6, Online Resource 1). MiRNAs and vectors were transfected with Lipofectamine 2000 (Life Technologies). After 48 h cells were collected for protein and RNA extraction. The 3′-untranslated region (UTR) of *NOTCH1* was amplified using the primers in Supplementary Table 5 (Online Resource 1) and cloned downstream of Renilla luciferase in the psiCHECK2 vector (Promega). Substitutions into the miR-200 and miR-34 binding sites of the *NOTCH1* 3′UTR were introduced by Quick-Change site-directed mutagenesis (Stratagene) (Supplementary Table 5, Online Resource 1). The firefly luciferase activity of psiCHEK2 (Promega) was used as a reference. Transfection was conducted in 24-wells plates. Each well was co-transfected with psiCHECK2 (400 ng) and miRNA precursor or negative control (30 pmol) (NC2, Life Technologies). Firefly and Renilla luciferase activities were measured 48 h after transfection using the Dual-Luciferase Report Assay (Promega). All experiments were replicated and performed at least in triplicate.

### Immunoblotting

Cells were collected from six-well plates using trypsin-EDTA and dissolved in lysis buffer (M-PER; Thermo Scientific) supplemented with complete protease (GE Healthcare) and phosphatase (Sigma-Aldrich) inhibitors. After electrophoresis and blotting, the primary antibodies (β actin 4967, Cell Signaling; NOTCH1 552466, BD Pharmingen; NOTCH1 C-20, Santa Cruz; Vinculin H-300, Santa Cruz) were incubated overnight at 4 °C. The peroxidase-conjugated anti-mouse or anti-rabbit antibodies were incubated for 1 h at room temperature and detected by chemiluminescence (Pierce ECL Western Blotting Substrate; Thermo Scientific), β-actin or vinculin normalized loading. The digitalized signals were quantified in the linear range of the scanner using ImageJ 64 software (http://imagejdocu.tudor.lu/).

## Results

### NOTCH signaling is the most significant pathway targeted by CNVs

**Fig. 1:**
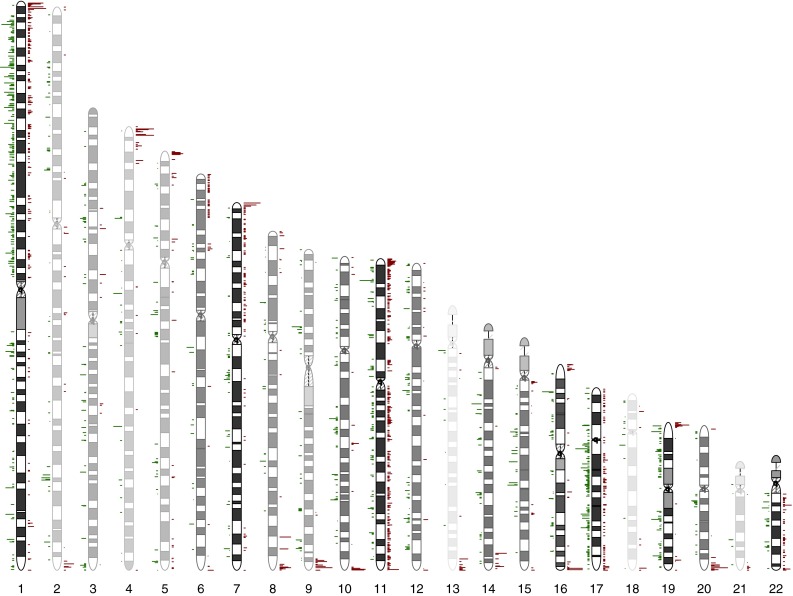
Positions of the paraganglioma-associated CNVs on the autosomal chromosomes. *Darker* ideograms highlight the chromosomes with higher CNV densities (e.g., 1p, 7p, 11p, 11q, 17p, 17q, 19p, 19q and 22q). *Green bars* to the left of the autosomal silhouettes indicate losses, *red bars* to the right gains. *Bar lengths* are proportional to the observed CNV frequencies. Notably, the telomeric regions show frequent gains, even in the chromosomes less densely affected by CNVs (e.g., chromosomes 2q, 4p, 5p, 13q, 18q)

*Leaf* analysis [41] of the data generated by the Illumina Omni 1 array (>10^6^ SNP) from 24 independent primary PGLs and paired blood from 23 patients (Supplementary Table 1, Online Resource 1) revealed a total of 19370 autosomal CNV calls (6777 in blood and 12593 in tumors). Figure 1 illustrates the chromosomal positions of the tumor-associated CNVs, obtained by subtracting the germline CNVs detected in the paired blood. Chromosomes 1p, 7p, 11p, 11q, 17p, 17q, 19p, 19q and 22q were more densely affected.

To highlight the genes and the molecular pathways most frequently targeted by CNVs, we first identified the top affected genes, i.e., those showing the highest level of CNV concordances among tumors (*P* < 0.01 by Fisher’s exact test). This highlighted 104 genes, of which 67 targeted by amplifications, 22 by deletions and 15 by both amplifications and deletions (Supplementary Table 7, Online Resource 1). None of these 104 genes has been previously associated with PGL. Some have been implicated in non-neoplastic diseases (e.g., *IDUA*, top amplified gene, associated to mucopolysaccharidosis type I) [53], some have unknown functions (e.g., *TMEM41B*, top deleted gene), some are involved in organogenesis and oncogenesis (e.g., *NOTCH1*, master regulator of differentiation and tumorigenesis) [50].

We then used the DAVID bioinformatics resources (http://david.abcc.ncifcrf.gov) to identify the enriched biological themes and the functional-related gene groups among the top 104 CNV-affected genes. Only the term “NOTCH signaling pathway”, which included *NOTCH1*, *HES5*, *JAG2*, *DVL1* and *CTBP1*, was statistically enriched after Bonferroni and Benjamini corrections (*P* = 0.0020 for both, Supplementary Table 8, Online Resource 1). *Leaf* analysis [41] indicated that the 5 NOTCH signaling-related genes were all amplified. In addition, 23 of the 48 partially redundant terms in the DAVID listing included *NOTCH1* or genes related to NOTCH signaling, although these terms were not statistically significant after Bonferroni or Benjamini corrections (Supplementary Table 8, Online Resource 1).

The copy number assignments obtained with *Leaf* analysis were orthogonally validated using NFMP-HPLC or qPCR for the 5 NOTCH signaling genes, for *IDUA* (top amplified gene), and for three deleted genes, *AKIRIN1*, *PHACTR4* and *SDHB*. Overall, the orthogonal validations of these CNV hits yielded reproducible results (average coefficient of variation: 8.17 %, range 0.17–17.4 %). In particular, the CNV and the NFMP-HPLC assays were concordant in 77.5 % (*NOTCH1*), 84.0 % (*DVL1*) and 95.6 % (*SDHB*) of the tested samples. CNV and qPCR yielded concordances of 82.6 % (*JAG2*), 67.4 % (*HES5*), 76.6 % (*CTBP1*), 72.3 % (*AKIRIN1*), 85.1 % (*IDUA*) and 100 % (*PHACTR4*). The overall average concordance was 82.3 %. The orthogonal validation data are provided in Appendixes 1 and 2, Online Resource 3).

### NOTCH1 mRNA is overexpressed in paraganglioma versus Jacobson’s nerve

**Fig. 2:**
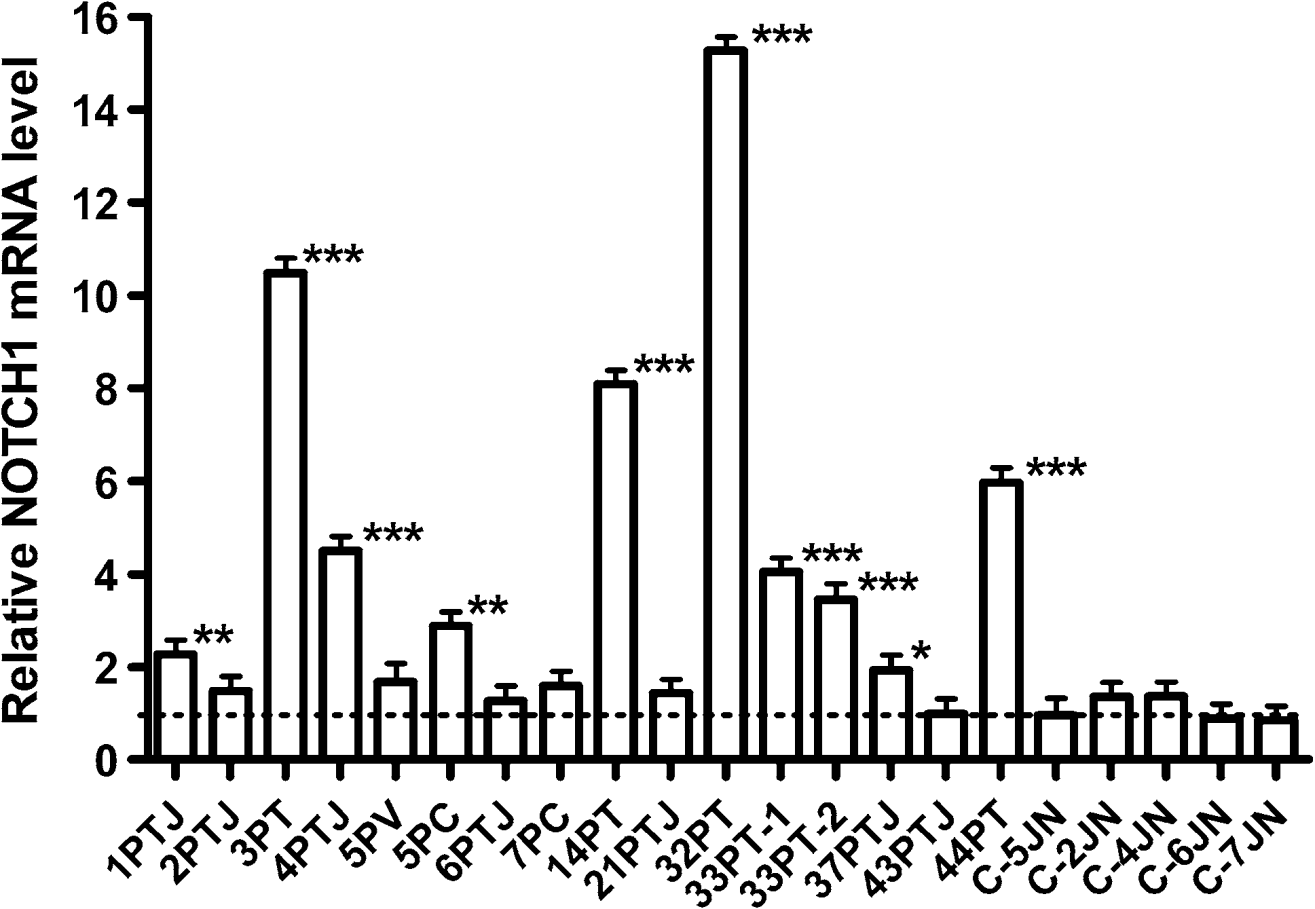
Relative *NOTCH1* mRNA levels measured by qRT-PCR in 16 paragangliomas relative to 5 Jacobson’s nerves. The *dashed line* indicates the average *NOTCH1* mRNA level of the 5 Jacobson’s nerve samples (C-JN) used as controls. Samples 33PT-1 and 33PT-2 are from different areas of the same tumor. The relative *NOTCH1* mRNA levels were calculated with the 2^−ΔΔCt^ method, using *ACTB* as reference. *Asterisks* indicate significantly higher *NOTCH1* mRNA levels after unpaired 2-tailed *t* test. *P < 0.05; **P < 0.01; ***P < 0.001. *Error bars* represent standard deviation

To obtain evidence of possible in vivo deregulation, we tested whether *NOTCH1* mRNA was overexpressed in PGLs compared to control JNs. *NOTCH1* expression was measured in 16 PGL samples (two, 33PT-1 and 33PT-2, from different areas of the same tumor) and compared to expression in 5 JNs. Overall, 10/16 PGL samples (56 %) showed significant *NOTCH1* overexpression, with relative mRNA levels ranging from 2.27 to 15.27 (Fig. 2). Four out of nine PGLs that demonstrated *NOTCH1* mRNA overexpression (i.e., 1PTJ, 4PTJ, 5PC, and 32PT) showed *NOTCH1* gains in the CNV analysis as well.

### Expression of NOTCH pathway proteins

**Fig. 3:**
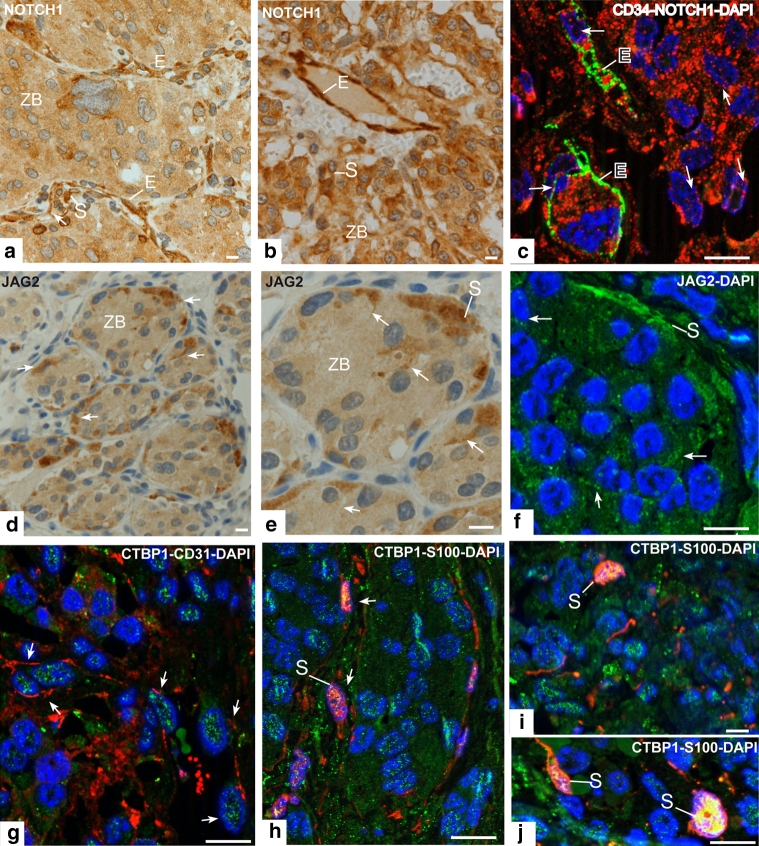
NOTCH1, JAG2 and CTBP1 immunolabeling in paraganglioma. **a**, **b** Exemplify NOTCH1 immunostaining in paraffin-embedded sections (different tumors). NOTCH1 is expressed in the three main PGL cell types, i.e., chief, sustentacular and endothelial (100 % for each cell type), with distinctly higher intensity in sustentacular (*S*) and, particularly, endothelial (*E*) cells. Apotome immunofluorescence, performed on single focal planes of frozen sections, highlights a punctate distribution of NOTCH1 (*red*) in the cytoplasm, along the nuclear profiles and inside the nuclei (*arrows*) of chief, sustentacular and endothelial cells, the latter identified by double labeling (*green*) with CD34 (**c**). Immunostaining on formalin-fixed, paraffin-embedded sections shows that JAG2 is diffusely expressed in the zellballen, with markedly higher intensity in the sustentacular cells, including their cytoplasmic processes (**d**, **e**, *arrows*). These JAG2 localizations are supported by Apotome immunofluorescence on frozen sections, which demonstrates punctate JAG2 labeling in the cytoplasm and along the plasma membranes of chief cells (*arrows*) and strong, diffuse cytoplasmic labeling of sustentacular (*S*) cells (**f**). Apotome immunofluorescence analysis of CTBP1 also shows high protein expression, with punctate pattern, in the cytoplasm and, more prominently, in the nuclei of all three PGL cell types (**g**–**j**). Notably, CTBP1 labeling appears accentuated in elongated nuclei of cells expressing the specific endothelial marker CD31 (**g**, *arrows*). Strong colocalizations of CTBP1 with S100 (*yellow*) are notable in the nuclei of sustentacular cells (**h**–**j**). *E* endothelial cells, *S* sustentacular cells, *ZB* zellballen. *Bars* 10 μm. Original single-channel grayscale images for the merged colocalizations of CTBP1 and S100 shown in **h**–**j** are illustrated in Supplementary Fig. 3 (Online Resource 2)

Supplementary Fig. 2 in Online Resource 2 shows the histoarchitecture of PGL. We investigated three proteins belonging to the NOTCH pathway highlighted by CNV analysis, i.e., NOTCH1 (receptor), JAG2 (ligand) and CTBP1 (signaling coregulator). IHC on FFPE sections indicated that NOTCH1 was expressed in the three main PGL cell types (Fig. 3a, b; Supplementary Table 3, Online Resource 1).

Apotome IF highlighted punctate NOTCH1 labeling in the cytoplasm, along the nuclear profiles and inside the nuclei of chief, sustentacular and endothelial cells, the latter co-expressing CD34 (Fig. 3c). IHC on FFPE sections showed that JAG2, undetectable in endothelia, was expressed in sustentacular cells, including their filamentous processes (Fig. 3d, e).

Apotome IF supported these observations (Fig. 3f) and also showed high CTBP1, with punctate pattern, in the cytoplasm and nuclei of chief, endothelial and sustentacular cells (Fig. 3g–j). Nuclear CTBP1 was accentuated in endothelial cells (identified by CD31 expression; Fig. 3g) and strongly colocalized with S100 in sustentacular cells (Fig. 3h–j, Supplementary Fig. 3, Online Resource 2).

The immunostaining for NOTCH1 and JAG2 in chief, sustentacular and endothelial cells was assessed by semiquantitative IHC on representative FFPE sections of 47 PGLs, including 21 tumors evaluated for CNV status at *NOTCH1* and *JAG2* (Supplementary Table 3, Online Resource 1). Diffuse NOTCH1 immunostaining in 100 % of each of the three main PGL cell types (chief, sustentacular and endothelial) was observed in all the PGLs examined (47/47), but the staining intensities differed significantly, in the following descending order: endothelial cells >sustentacular cells >chief cells (Table 1; Fig. 3a, b; Supplementary Table 3, Online Resource 1). In fact, NOTCH1 staining intensity resulted higher in endothelial cells versus both chief cells and sustentacular cells (in both cases *P* < 0.0001 by independent sample *t* test, Table 1), and sustentacular cells were also more intensely stained than chief cells (*P* = 0.0067, Table 1). JAG2 was expressed in 41 out of the 45 PGLs in which JAG2 IHC could be performed (91 %). JAG2 immunostaining intensity was significantly higher in sustentacular relative to chief cells (*P* = 0.0061, Table 1; endothelial cells resulted JAG2 negative). Notably, high levels of NOTCH1 and JAG2 immunostaining were observed also in the PGLs that did not show CNVs at the relevant genes (Supplementary Table 3, Online Resource 1).

**Table 1:** Independent samples *t* test analysis of NOTCH1, JAG2 and BCL2 immunostaining intensity levels in the three main paraganglioma cell types (chief cells, sustentacular cells and endothelial cells)

Cell type	Gene	Mean ± SE	*P*
	NOTCH1		
Chief		1.85 ± 0.069	0.0067
Sustentacular		2.00 ± 0.08	
Chief		1.85 ± 0.069	<0.0001
Endothelial		2.78 ± 0.061	
Sustentacular		2.00 ± 0.08	<0.0001
Endothelial		2.78 ± 0.061	
	JAG2^a^		
Chief		0.95 ± 0.056	0.0061
Sustentacular		1.18 ± 0.09	
	BCL2		
Chief		0.17 ± 0.09	
Sustentacular		1.11 ± 0.09	<0.001
Chief		0.17 ± 0.09	
Endothelial		0.8 ± 0.09	<0.001
Sustentacular		1.11 ± 0.09	<0.001
Endothelial		0.8 ± 0.09	

^a^JAG2 was not expressed in endothelial cells

**Fig. 4:**
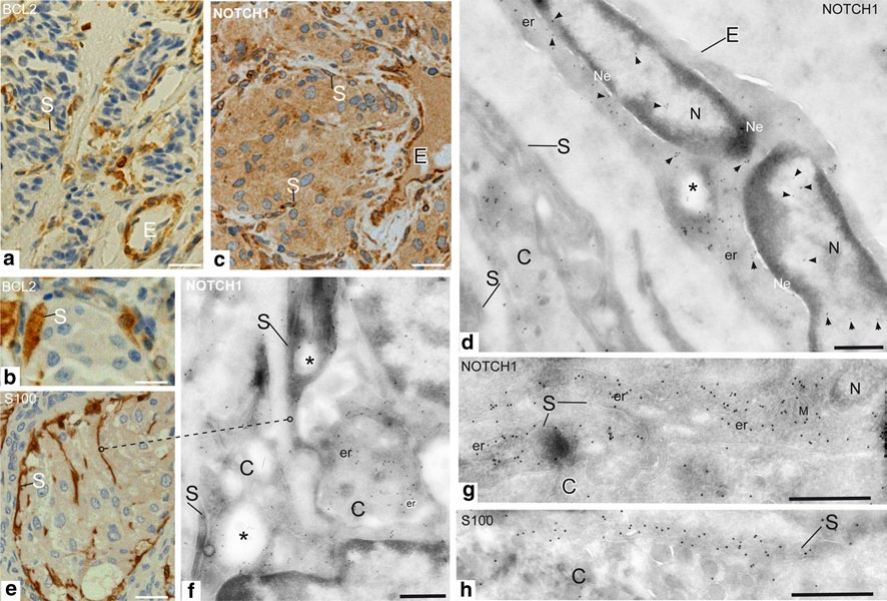
BCL2 and NOTCH1 immunolabeling in sustentacular and endothelial cells. Endothelial (*E*) and sustentacular (*S*) cells are intensely labeled with BCL2 (**a**, **b**). The higher level of NOTCH1 immunostaining in these same cell types, evidenced by immunohistochemistry on paraffin sections (**c**), is supported by cryo-immunoelectronmicroscopy, which shows NOTCH1 gold along the endoplasmic reticulum, nuclear membrane and endosomes of endothelial cells (**d**). Some labeling is also present within the nuclei (**d**). The sustentacular cells and their filamentous processes, that deeply penetrate within the tumor cell nests, are identified by S100 immunolabeling using both immunohistochemistry and cryo-immunoelectronmicroscopy (**e**, **h**). These processes are densely labeled with NOTCH1 gold (**d**, **g**), that localizes particularly along plasma membrane contacts with chief cells (**f**). Where sustentacular processes envelope chief cells, NOTCH1-labeled endosomal structures are observable in both cell types (*asterisks*, **f**). *N* nucleus, *Ne* nuclear envelope, *er* endoplasmic reticulum, *S* sustentacular cell, *C* chief cell. *Bars* **a**–**c**,** e** = 20 μm,** d**,** f**–**h** = 1 μm

The same PGLs were immunostained for synaptophysin, chromogranin A, S100, Ki67, vimentin and BCL2. As expected [32], all the tumors strongly expressed synaptophysin (both sustentacular and chief cells), chromogranin A (mainly chief cells) and S100 (mainly sustentacular cells). BCL2 resulted significantly higher in the sustentacular relative to both the endothelial and the chief cells (*P* < 0.001) and also in the endothelial versus the chief cells (*P* < 0.001) (Table 1; Fig. 4a, b). Given the substantial uniformity of the NOTCH1 and JAG2 expression patterns in the tested PGLs, no correlations emerged between the immunostaining for these proteins and the other variables (Supplementary Table 3, Online Resource 1 and data not shown).

### Germline *SDH* mutations and SDHB immunohistochemistry versus NOTCH1/JAG2 expression

Considering that NOTCH signaling activation could be related to *SDH* gene defects, the PGL cases were analyzed for germline *SDH* mutations and/or tumor-associated loss of SDHB expression, a surrogate marker for mutations in any of the known PGL-related mitochondrial complex II genes. Germline *SDH* gene mutation analyses, performed in 34 cases, identified 13 mutation carriers, furthermore CNV analysis identified a large germline deletion/rearrangement of *SDHB* in one additional case (8PTJ, confirmed by NFMP-HPLC). The overall frequency of germline mutations in *SDHB*, *SDHC*, *SDHD* and *SDHAF2* was 14/35 (40 %); single gene mutation frequencies are detailed in Supplementary Table 9 (Online Resource 1). The germline *SDH* mutation frequencies in the clinically relevant subsets of the tested PGLs (Supplementary Table 10, Online Resource 1) were in substantial agreement with the literature data [6, 8, 25, 39, 47]. *SDH* mutation status resulted associated to negative SDHB IHC, with 12 SDHB-negative tumors out of 14 tumors from identified *SDH* gene mutation carriers (85.7 %), versus 6 SDHB-negative tumors out of 20 tumors that tested negative for *SDH* genes mutations (30 %) (*P* = 0.0019 by Fisher’s exact test; Supplementary Table 9, Online Resource 1).

**Fig. 5:**
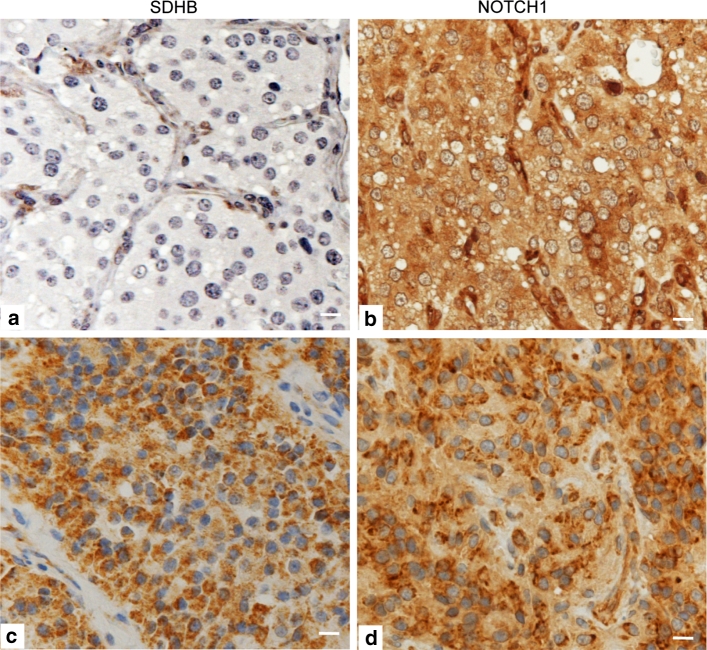
SDHB versus NOTCH1 immunostaining. A paraganglioma showing tumor-associated loss of SDHB immunostaining (**a**) is compared to a paraganglioma with positive SDHB immunostaining (**d**). Both tumors show intense and diffuse NOTCH1 labeling (**b**, **d**). *Bars* 10 μm

Overall, 21 out of the 45 PGLs that were qualitatively adequate for SDHB IHC (46.6 %) resulted SDHB negative (Supplementary Tables 2 and 10, Online Resource 1). Differences in NOTCH1 or JAG2 IHC intensities between the SDHB-positive and the SDHB-negative PGLs were not significant by two-tailed Student’s *t* test (Fig. 5; Supplementary Tables 2, 3 and 11, Online Resource 1).

### Cellular and subcellular localizations of NOTCH1 by cryo-IEM

**Fig. 6:**
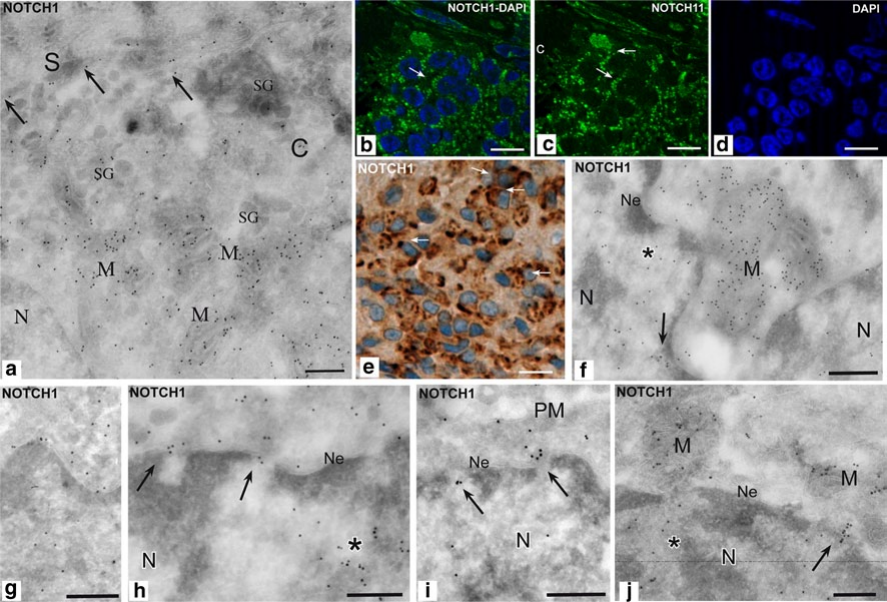
Subcellular localizations of NOTCH1 in paraganglioma. Cryo-immunoelectronmicroscopy shows that NOTCH1 strongly labels mitochondria, with preferential localization of gold particles on mitochondrial membranes (**a**, **f**, **j**). This explains the punctate cytoplasmic NOTCH1 staining observed by Apotome immunofluorescence in frozen sections (**b**–**d**) and the perinuclear NOTCH1 labeling often evidenced by immunohistochemistry in paraffin-embedded sections (**e**). Some NOTCH1 labeling is also observed in discrete nucleoplasmic areas (*asterisks*) and along the nuclear membranes (**f**, **g**), mostly near nuclear pores (**h**–**j**). Notably, the NOTCH1-labeled mitochondria tend to concentrate near the nuclear membrane (**a**–**f**), particularly in correspondence of nuclear pores and of NOTCH1-labeled nucleoplasm (**f**–**j**). As shown before, NOTCH1 gold particles label plasma membrane contacts between sustentacular cells and chief cells (**a**). *C* chief cell, *N* nucleus, *Ne* nuclear envelope, *M* mitochondrion, *S* sustentacular cell, *SG* secretory granules. *Bars* **b**–**e** = 10 μm,** a**,** f**–**j** = 1 μm

Cryo-IEM confirmed high NOTCH1 expression in the sustentacular cells and their elongated processes, identified by S100 labeling (Fig. 4c–h). Notably, NOTCH1 was detected in early endosomal structures (Fig. 4f) and along the plasma membrane, particularly at contact sites between sustentacular processes and chief cells (Figs. 4f, g; 6a). NOTCH1 labeling was also evident in endothelial cells, along the nuclear and the plasma membranes, in endosomes and in the endoplasmic reticulum (Fig. 4d).

Cryo-IEM provided ultrastructural support for interpreting the punctate NOTCH1 cytoplasmic staining observed by IF (Fig. 6b–d) and the perinuclear labeling detected by IHC (Fig. 6e). In fact, regardless of the tumor cell type, NOTCH1 strongly labeled the mitochondria, with preferential localization along the mitochondrial membranes (Fig. 6a, f, j). Some labeling was also observed in discrete nucleoplasmic areas and in correspondence of the nuclear membranes, particularly near nuclear pores (Fig. 6f–j). Interestingly, the NOTCH1-labeled mitochondria tended to concentrate in perinuclear position (Fig. 6a, f, j), being in some cases closely associated to the nuclear membrane, in correspondence of nuclear pores and of NOTCH1-labeled nucleoplasm (Fig. 6f, j).

### miRNAs controlling NOTCH are deregulated in PGL

Since tumors with or without evidence of CNVs at NOTCH1 and JAG2 showed overexpression of the relevant gene products, we hypothesized that miRNAs could provide a complementary mechanism of NOTCH signaling deregulation in PGL. To identify miRNAs that could have a role in PGL, we performed genome-wide miRNA profiling in 14 PGL samples from 13 independent tumors (two distinct areas from tumor 33PT were analyzed in this assay) versus JN control pools (Supplementary Tables 1 and 4, Online Resource 1). We identified 16 miRNAs significantly (*P* < 0.05) downregulated and 3 miRNAs significantly upregulated in PGLs (Table 2).

**Table 2:**
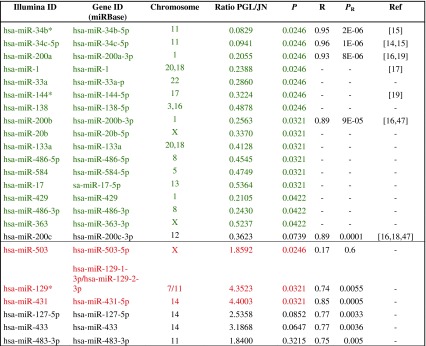
MiRNAs differentially expressed in paragangliomas versus Jacobson’s nerves

The Table shows 16 miRNAs significantly (*P* < 0.05) downregulated (*green*) and three significantly upregulated (*red*). MiRNAs whose expression resulted not significantly different in paragangliomas (*PGL*) versus Jacobson’s nerve (*JN*) were used to validate the Illumina array by qRT-PCR (*black*). The Pearson coefficient of correlation (*R*) and its *P* value (*P*_R_), calculated with the qRT-PCR data, are indicated. The last column (*Ref*) lists the reference studies that link the NOTCH pathway to the considered miRNA

Next, we validated the miRNA array data by qRT-PCR in 10 PGLs, 1 JN and SH-SY5Y cells. The Pearson correlation coefficient (R) was calculated for 11 miRNAs (5 downregulated and 6 upregulated), chosen irrespectively of the *P* values resulting from microarray analysis (Table 2; Supplementary Fig. 4a, b, Online Resource 2). The Pearson coefficient of correlation between microarray and qRT-PCR expression values ranged between 0.17 and 0.95 (median: 0.85), supporting the reliability of the microarray output (Table 2). Notably, miR-503-5p, the most significantly upregulated miR in our assay, showed low, non-significant correlation (*R* = 0.168; *P* = 0.6) and was therefore excluded from further studies, although its concordance with the upregulation data analysis was greater than 80 %.

Remarkably, five of the most downregulated miRNAs, including miR-34b-5p, miR-34c-5p, miR-200a-3p, miR-200b-3p, and miR-200c-3p, were linked to the NOTCH pathway, although in different cellular contexts [3, 4, 26, 31, 70]. None of these miRNAs was affected by CNVs.

Most of the tested PGLs showed marked downregulation of miR-34b-5p, miR-34c-5p, miR-200a-3p, miR-200b-3p, and/or miR-200c-3p (Supplementary Figure 4a, Online Resource 2), often co-occurring with genomic amplifications of NOTCH pathway genes, with the exception of 32PT, that had no or modest downregulation of the miRNAs targeting the NOTCH pathway and showed amplification of *NOTCH1*, *JAG2*, *CTBP1*, *HES5*, *DVL1* (CNVs detailed in Appendix 1, Online Resource 3).

### The miR-200 and miR34 gene families target NOTCH1 and sensitize primary PGL cells to cell death

The *NOTCH1* 3′UTR contains predicted binding sites for the miR-34 (miR-34ac/34c-5p/34b*/449abc/449c-5p) and miR-8 (miR-200bc/429/548a) gene families (http://www.targetscan.org/vert_61/), that include miRNAs that we found downregulated in PGLs (Supplementary Fig. 5, Online Resource 2). After comparing the list of the predicted miRNAs targeting *NOTCH1* and the list of the miRNAs downregulated in PGL, we decided to investigate *NOTCH1* as direct target of miR-34c-5p and miR-200b-3p in the SH-SY5Y background. Because of the sequence homology, we also considered miR-34b* (miR-34b-5p) and miR-200a (miR-200a-3p), both downregulated, although not predicted to target the *NOTCH1* 3′UTR. In addition, we studied miR-200c (miR-200c-3p), because it appears to target NOTCH pathway components, such as *JAG1* and the mastermind-like coactivators *MAML2* and *MAML3* [4], although its downregulation in PGLs did not reach statistical significance (*P* = 0.0739). None of these miRNAs map to the CNV regions detected in the tested PGL series (data not shown).

**Fig. 7:**
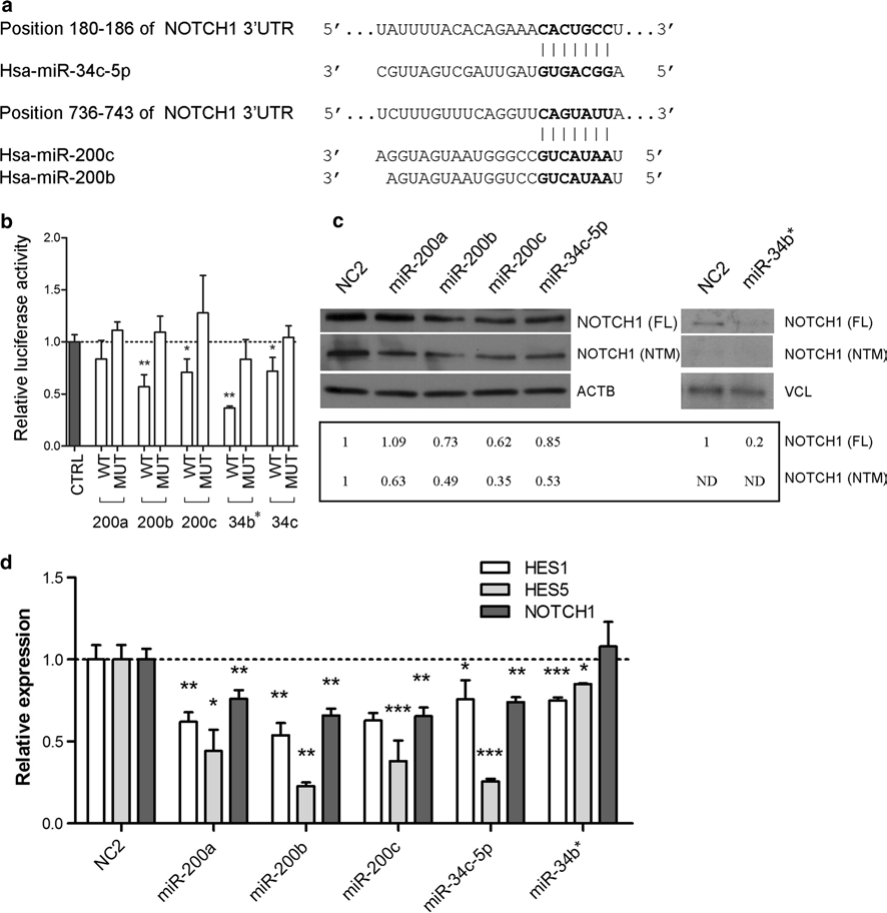
NOTCH1 is target of miR-200s and miR-34s. **a** Putative binding sites of miR-34b-5p, miR-200b and miR-200c in NOTCH1 3′UTRs (TargetScan). *Asterisks* indicate nucleotides substituted in miR-34s and miR-200s predicted target sites to perform the luciferase assays. **b** NOTCH1 3′UTRs regulate luciferase activity dependent on miR-200b, miR-200c, miR-34b* and miR-34c-5p in SH-SY5Y cells (*WT* wild type, *MUT* mutant, *P* P value). Renilla luciferase activity was normalized on the firefly luciferase activity of the pSICHECK2 vector. **c** Western blot analysis of NOTCH1 (BD, 552466 and Santa Cruz, sc-6014R), β-actin (ACTB) and vinculin (VCL) after microRNAs transfection in SH-SY5Y cells; the full-length (FL, ~300 kDa) and NOTCH1 transmembrane fragment (NTM, ~120 kDa) are indicated. Cells were collected at 48 h from miRNA transfection. Normalization with densitometric analysis is shown. **d** Real-time quantitative reverse transcription PCR analysis for the NOTCH1 transcriptional target genes HES1, HES5 and NOTCH1 (*P < 0.05; **P < 0.005; ***P < 0.0005, *ND* not detectable)

To test direct interactions with the *NOTCH1* 3′UTRs, the predicted wild-type and mutant miR-200s and miR-34s target sites of the 3′UTR-*NOTCH1* RNA were cloned downstream of the psiCHECK2 Renilla reporter. Then, either the wild type or the mutant forms of the psiCHECK2 3′UTR-*NOTCH1* vectors were co-transfected with miRNA mimics into SH-SY5Y cells. Compared to controls, significant reductions in the luciferase activity of the psiCHECK vector carrying the wild-type *NOTCH1*–3′UTR were registered with miR-200b (~43 %), miR-200c (~29 %), miR-34b* (~64 %) and miR-34c (~29 %), but not with miR-200a, whereas in the mutated clones luciferase activity was not significantly downregulated by the tested miRNAs (Fig. 7a, b).

To further confirm *NOTCH1* as target of miR-200b, miR-200c, miR-34b*and miR-34c, NOTCH1 protein levels were assessed in SH-SY5Y cells after miRNAs transfection. Expression of the full-length NOTCH1 was reduced by miR-200b (~27 %), miR-200c (~38 %), miR-34b* (~80 %) and miR-34c (~15 %) at 48 h from transfection, but, again, not by miR-200a (Fig. 7c). However, cleaved active NOTCH1 (NTM) was reduced also after enforced expression of miR-200a in SH-SY5Y cells (Fig. 7c), suggesting the involvement of this miR in NOTCH1 regulation, as already proposed [58, 70]. To corroborate these results, we next assessed the mRNA levels of two transcriptional targets of NOTCH1 (*HES1* and *HES5*) at 48 h from miRNA mimics transfection. In all the RNA samples tested *HES1* and *HES5* significantly decreased (Fig. 7d). Since there are no predicted miR-200s and miR-34s binding sites within the 3′UTR of *HES1* and *HES5*, this was most likely due to lower NOTCH1 level.

**Fig. 8:**
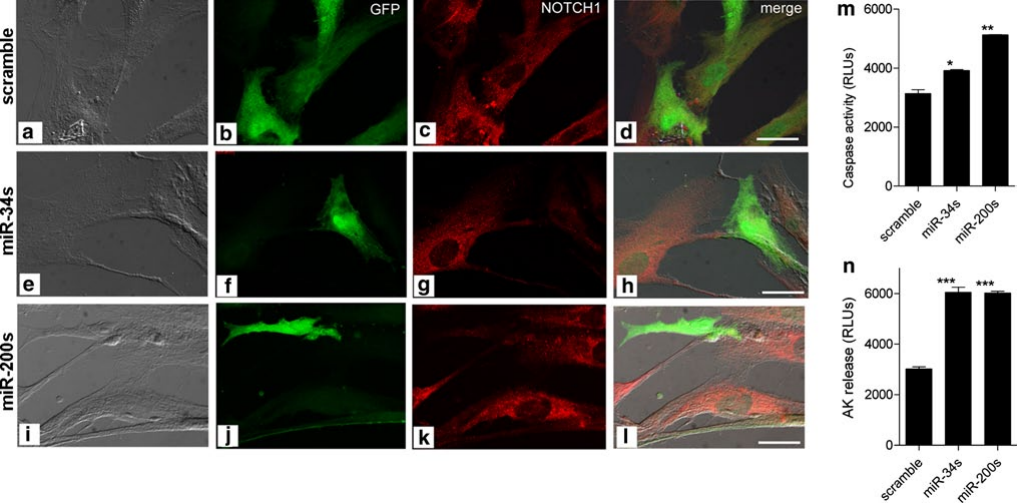
Primary PGL cells infected with lentiviruses transducing miR-200s and miR-34s show downregulation of NOTCH1 expression and increased markers of cytotoxicity and death. Primary paraganglioma cell cultures PTJ64p were infected with lentiviral vectors transducing scramble miR control (scramble, **a**–**d**, *bar* 10 μm), miR-34 cluster (miR-34s, **e**–**h**, *bar* 10 μm) and miR-200 cluster (miR-200s, **i**–**l**, *bar* 20 μm). **a**, **e**, and **i** show Apotome light microscopic views of the exemplified cells. By Apotome immunofluorescence, ectopic expression of the miR-34s and miR-200s results in clear reduction of the NOTCH1 protein signal (*red* g for miR-34s, k for miR-200s), only in cells expressing green fluorescent protein (GFP, *green*), which marks lentivirally infected cells (**f** miR-34s; **j** miR-200s) and not in GFP-negative (i.e., non-infected) cells present in the same culture. No differences in NOTCH1 expression are apparent in cells infected with scramble miR control (GFP-positive) versus non-infected cells (GFP-negative) in the same culture (**b** scramble GFP; **c** scramble NOTCH1). Merged immunofluorescence signals for GFP (*green*) and NOTCH1 (*red*) are shown in **d** (scramble miR control), **h** (miR-34s) and **l** (miR-200s). **m** Shows the results of the caspase 3/7 assay (Promega) on the same cells. **n** Shows the citotoxicity assay of PTJ64p cells infected with lentiviral vector transducing scramble miR control, mir-34s and miR-200s, measured by the adenylate kinase activity (Lonza) in the medium of cultured cells. *P < 0.05; **P < 0.01; ***P < 0.001. *Error bars* represent standard deviation

Next, we examined whether the miR-200s and the miR-34s could induce cell death. Infection with a GFP lentiviral vector expressing either the miR-200s or the miR-34s effectively rescued the expression of the candidate miRNAs in PTJ64p, a primary human PGL cell culture that we developed from a tumor showing downregulation of the miR-34s and miR-200s by qRT-PCR (Supplementary Fig. 6a, b Online Resource 2). More than 75 % of the cultured cells became GFP-positive after infection. Ectopic expression of the relevant miRNAs in PTJ64p (Supplementary Fig. 6b, Online Resource 2) resulted in a clear reduction of NOTCH1 protein expression by IF, indicating that the miRNAs transduced by the lentivirus vector were functional (Fig. 8a–l). Importantly, the expression of the transduced miRNAs in PTJ64p was associated with higher caspase-3 activity (*P* = 0.0284 for the miR-34s; *P* = 0.0043 for the miR-200s) and higher adenylate kinase release (*P* = 0.0002 for the miR-34s; *P* < 0.0001 for the miR-200s), as compared to the scramble control (Fig. 8m, n), suggesting that the candidate miRNAs sensitize primary human PGL cells to death.

## Discussion

The genes involved in genetic susceptibility to PGL have been extensively studied, but little is known about the molecular pathways that drive PGL tumorigenesis at the somatic level [9, 18]. Previous investigations, that utilized low density approaches, such as comparative genomic hybridization (CGH) and loss of heterozygosity analysis, revealed frequent deletions of chromosome arms 1p, 3q and 22q and multiple minimal overlapping regions of deletion in at least 16 chromosomes, particularly 1p, 3q, 11p/q, 17p and 22q, while the most common minimal regions of gain were in 1q, 7p, 12q and 19p [18, 52, 54]. The existence of recurrent losses and gains in several chromosomes suggests that multiple genes are inactivated or activated in PGLs. To shed light on the genes and genetic pathways implicated in head and neck PGL, we first relied on high-density genome-wide CNV analysis. Our high-resolution analysis was in substantial agreement with the CGH- and LOH-based literature and revealed multiple recurrent losses or gains in several autosomes, with 1p, 7p, 11p, 11q, 17p, 17q, 19p, 19q and 22q more densely affected.

Following a gene-centric approach, we next identified 104 genes that were more frequently (*P* < 0.01) affected by CNVs. Gains were more frequent than losses in this top genes list. The genes were functionally diverse and had never been linked to PGL.

In the present work we focused on the most over-represented functional gene cluster, “NOTCH signaling” pathway, identified by submitting the list of the 104 genes with highly recurrent CNVs to the DAVID tool (http://david.abcc.ncifcrf.gov). Canonical NOTCH signaling is a highly conserved contact-dependent intercellular signaling mechanism which, interacting with other molecular networks depending on cell/tissue contexts, controls a diversity of proliferation/differentiation processes, including embryofetal neurogenesis, gliogenesis and vasculogenesis, as well as physiological or pathological neoangiogenesis and glial homeostasis in the central and peripheral nervous systems [46, 50, 61, 69]. NOTCH signaling has multiple fundamental roles in cancer, where, among other activities, critically regulates stem-like cancer cells and contributes to hypoxia responses, epithelial-mesenchymal transition (EMT), angiogenesis and invasiveness [50, 57]. Both normal neural stem cells and stem-like cells of neural tumors require NOTCH for modulation of self-renewal versus glial, neuronal and endothelial differentiation [57]. Notably, NOTCH dysregulation is implicated in highly angiogenic neural tumors, including glioblastoma and medulloblastoma, the leading intracranial cancers in adults and children, respectively [16, 23, 49, 57], as well as neuroblastoma, major neuronal cancer of childhood, which, as PGL and pheochromocytoma, is of paragangliar origin [10].

In the PGL series genotyped for CNVs the high statistical significance of the “NOTCH signaling pathway” rested on five genes targeted by recurrent amplifications, i.e., *NOTCH1* (9q34.3), *JAG2* (14q32), *HES5* (1p36.32), *DVL1* (1p36), and *CTBP1* (4p16). These CNVs were confirmed using orthogonal assays. *NOTCH1*, prototype of a family with four developmentally regulated and tissue-specific members (*NOTCH1/4*), encodes a transmembrane receptor that, after interaction with cognate ligand(s) expressed on adjacent cells, is converted into a transcription factor [50]. Signal transduction is initiated by consecutive proteolytic cleavages that free the nuclear-bound NOTCH1 intracellular domain (NICD1) [50]. NICD1 then forms enhancer complexes with tissue-specific transcriptional activators. NOTCH1 has been shown to have oncogenic roles in glial tumors [57] and its activation may be triggered by several mechanisms, including rearrangements and activating mutations [2].

The other NOTCH1 pathway genes targeted by frequent copy number gains illuminate a molecular context that may constrain NOTCH signaling towards biological effects relevant for PGL tumorigenesis. *JAG2* [38], one of the five canonical activators of NOTCH1, is hypoxia-dependent and correlates with EMT and invasion [48]. *HES5*, member of the HES (hairy enhancer of split) family of transcription factors, is a well-characterized transcriptional target of the NICD1 enhancer complex, implicated in neural stem cells induction [22]. DVL1 modulates NOTCH stability via GSK-3 inhibition and, by sustaining Wnt/beta-catenin signaling, cooperates with the NOTCH pathway in promoting the proliferation and differentiation of neural stem cells [17]. Moreover, DVL1 specifically increases NOTCH signaling in endothelia, inducing sprouting and altering vascular differentiation [13]. CTBP1, a coregulator implicated in cancer and EMT [12], links the transcriptional effects of NICD to oxygen and nutrients [60] and to sprouting angiogenesis [51]. In sum, the five NOTCH signaling genes amplified in PGL modulate interconnected pathways implicated in the development and cross-talk of neural and endothelial cells.

Analysis of NOTCH1 and JAG2 expression by IHC in a series of 47 FFPE PGLs, including most of the cases genotyped by CNV analysis and a case for which we obtained primary cell cultures, provided a further level of evidence supporting the key role of NOTCH signaling in PGLs. These studies were complemented with IF and cryo-IEM, to define the cellular and subcellular localizations of the gene products and the relationships with known PGL-related markers. IHC demonstrated NOTCH1 immunostaining in all the PGLs analyzed, regardless of the individual or clinicopathological characteristics. Furthermore, NOTCH1 and JAG2 were highly expressed in tumors with or without evidence of CNV at the respective loci. This suggested that NOTCH1 signaling is a fundamental PGL pathway and that its activation may involve genomic amplification along with other mechanisms. Importantly, within tumors, NOTCH1 and JAG2 immunostaining was significantly correlated to cell type, being NOTCH1 higher in sustentacular relative to chief cells and highest in endothelial cells, while JAG2, undetectable in the endothelium, was particularly evident in sustentacular cells. In this respect the filamentous processes, characteristic of this glial cell type, are predicted to greatly increase the JAG2-expressing plasma membrane surface. Furthermore, these processes establish multiple contacts with the plasma membranes of other cells, even at remarkable distance. Thus, sustentacular cells might amplify JAG2-dependent, contact-mediated NOTCH1 activation in the PGL microenvironment. By cryo-IEM membrane contacts between sustentacular and chief cells demonstrated immunoultrastructural evidence of NOTCH1 internalization in both cell types. Remarkably, JAG2 immunostaining paralleled the distribution of S100, a Ca(2+)-binding protein highly expressed in sustentacular cells, that performs pro-inflammatory and trophic functions and suppresses P53-dependent apoptosis [35]. This suggests that sustentacular cells may “nurse” chief cells with JAG2 in an S100-modulated microenvironment. A major role of sustentacular cells in PGL is consistent with the physiologic rat carotid body model, where the sustentacular population includes the stem cell component of the paraganglion [44].

As outlined above, cooperative signaling involving NOTCH1, JAG2, DVL1 and CTBP1 may represent a key pro-angiogenic mechanism in PGL. In glioblastoma, NOTCH signaling was proposed to promote endothelial trans-differentiation of tumor cells [16, 23, 57]. A similar mechanism might play a role in PGL, a possibility that needs to be tested in further studies. The hypothesis would be consistent with evidence, provided in this study, that PGL-associated endothelia are morphologically atypical, closely juxtaposed to or intimately admixed with sustentacular cells and positive for the cell surface sialomucin CD34, an endothelial progenitor marker associated with angiogenesis and migration [55]. Moreover, the highest levels of immunolabeling for NOTCH1 and for CTBP1, a coregulator implicated in NOTCH-induced angiogenesis [51], were observed in the nuclei of sustentacular and endothelial cells. These cells were also strongly positive for BCL2, a transcriptional target of the NOTCH pathway [50], which characterizes developmental and tumor-associated neoangiogenic endothelium and is implicated in the cross-talk between endothelial and cancer cells [28]. The immunoultrastructural localizations of NOTCH1, revealed by cryo-IEM, included mitochondria and nuclei, key subcellular sites of NICD1 signaling [45]. These localizations were observed in sustentacular, chief and endothelial cells, supporting NOTCH1 activation in all three cell types. The strong mitochondrial immunolabeling, mostly associated with the organelle membrane, is particularly interesting, as NICD1 was previously shown to inhibit BAX multimerization, thus upregulating resistance to apoptosis by nutrient-deprivation and oxidative stress [45]. Furthermore, in sustentacular and endothelial cells a pro-survival action of NICD1 would be boosted by the concomitant overexpression of BCL2, observed in this study. A key function of mitochondria in intracellular NOTCH1 trafficking, as proposed by Lee et al. [33], is supported by our ultrastructural findings in PGLs, strongly suggesting mitochondrial-nuclear shuttling of NICD1 [33].

Overexpression of the NOTCH signaling-related genes *JAG1* and *HES1* has been previously reported in association with *VHL*- and *SDHx*-related PGLs, that typically manifest a pseudo-hypoxic signature [7, 37]. Therefore, we addressed the question of whether NOTCH signaling activation was a general feature of the studied head and neck PGLs, or was particularly associated to cases with constitutional *SDH* gene defects. No differences in NOTCH1 and JAG2 protein expression were observed in the subset of PGLs from ascertained germline *SDH* mutation carriers compared to cases putatively negative for constitutional *SDH* mutations. Furthermore, we found no differences in NOTCH1 and JAG2 protein expression in the subset of PGLs showing SDHB stain loss, indicative of mutations in *SDH* subunit genes [19, 63], compared to the SDHB-positive subset. Based on these data we propose that NOTCH signaling activation is a basic feature of head and neck PGL, independent from the presence or absence of constitutional *SDH* gene defects.

Adler et al. [1] reported that treatment with histone deacetylase inhibitors, that upregulate NOTCH signaling, results in decreased growth and hormonal secretion in PC12 rat pheochromocytoma cells. This study is not necessarily in contrast with our data. In fact, it is well known that NOTCH1 is expressed in neural crest paragangliar progenitor cells [27]. In the presently studied human head and neck PGLs, the highest levels of NOTCH1, JAG2 and CTBP1 were found in cells of endothelial and/or glial phenotype. Thus, NOTCH signaling could preferentially drive the growth of these PGL-associated cell types.

Copy number variation (CNV) and immunomorphological data concur in supporting core role(s) for NOTCH1 and related genes, yet the evidence of protein overexpression also in cases with no proof of relevant CNVs implies that independent mechanisms back up or replace genomic changes as drivers of NOTCH activation. Given that translation of *NOTCH* mRNAs is negatively regulated by miRNAs [26, 34], we hypothesized that miRNA-based mechanisms could contribute to NOTCH1 deregulation in PGL. To identify candidate miRNAs we relied on genome-wide miRNA profiling of PGLs relative to structurally normal JN, the tissue of origin of tympanic PGL [32]. We identified a microRNA signature strictly correlated to the regulation of NOTCH1 and of its signaling pathway in PGL. We proved that the miR-200s and miR-34s directly target NOTCH1 and that miR-200a indirectly influences the NOTCH pathway in SH-SY5Y cells. These microRNAs are strictly correlated to the EMT and P53 pathways [20, 30] and their downregulation allows angiogenic responses [70]. Importantly, the miR-34, in addition to targeting NOTCH1, directly inhibits BCL2 translation [68]. Thus, in PGL, downregulation of the miR-34 could contribute to the concomitant overexpression of NOTCH1 and BCL2, observed in the sustentacular and in the endothelial cells. Notably, we show that ectopic overexpression, by lentiviral transduction, of the miR-200s and miR-34s effectively downregulates NOTCH1 in primary cell cultures of human tympano-jugular PGL and significantly increases caspase activity and adenylate kinase release. This suggests that forced downregulation of NOTCH signaling sensitizes primary human PGL cells to death.

In a previous study, Tombol et al. [62] reported that NOTCH signaling is one of the top canonical pathways potentially targeted by miRNA-based transcriptional repression in recurring pheochromocytomas. This observation is in contrast with our data, but a number of possibilities may account for the apparent divergence: (1) different from PGLs, pheochromocytomas are mostly chromaffin; (2) in the study of Tombol et al. [62] the differences in miR expression were estimated comparing distinct tumor subsets, whereas we compared our head and neck PGLs to JN, a histogenetically relevant normal tissue control; (3) the miRNA-affected pathways were only bioinformatically predicted in pheochromocytomas, whereas we performed functional studies to validate our findings.

The mechanisms underlying the reduced expression of the five presently reported miRNAs regulating NOTCH1 remain unclear. As none of these miRNAs were affected by CNVs, altered epigenetic regulation, such as aberrant DNA methylation or histone modifications, could represent a mechanism implicated in their downregulation, a possibility that will have to be investigated in future studies. Various other factors might play a role. JAG2, overexpressed in sustentacular cells, suppresses the miR-200 family in a murine model of metastatic adenocarcinoma [70]. The S100 proteins, also overexpressed in sustentacular cells, modulate P53 activity [35], and P53 downregulation would bring down the miR-34s and the miR-200s [30]. It should be noted that most of the tumors with marked downregulation of miRNAs targeting the NOTCH pathway showed also genomic amplifications of molecules in the same pathway. Therefore, it appears that both CNVs and miRNAs play a synergistic role in NOTCH pathway upregulation.

In conclusion, whole-genome CNV analysis, miRNA profiling and immunomorphology converge in indicating that a NOTCH signaling axis involving angiogenic modulators is commonly activated in PGL. NOTCH1 signaling appears upregulated in all the three main PGL cell types, sustentacular (glial), chief (neuroendocrine) and endothelial, possibly with a leading role of sustentacular cells, which overexpress ligand and receptor. NOTCH1 signaling is likely to be a key player in organoid PGL tumorigenesis and could be implicated in the resistance to radiotherapy and anti-angiogenic agents shown by this tumor type [23, 66]. The effects of NOTCH blockage should be further tested in in vitro and in vivo PGL models.

## Electronic supplementary material

Below is the link to the electronic supplementary material.

Supplementary material (PDF 282 kb)

Supplementary material (PDF 7790 kb)

Supplementary material (PDF 29 kb)
