# Supplementary material for: Integrative genetic, epigenetic and pathological analysis of paraganglioma reveals complex dysregulation of NOTCH signaling
Source: Acta Neuropathol. 2013 Aug 18;126(4):575–94. doi: 10.1007/s00401-013-1165-y (PMC3789891; doi:10.1007/s00401-013-1165-y)
Supplement: Supplementary file 1 — Supplementary material (PDF 282 kb) [file 401_2013_1165_MOESM1_ESM.pdf]

**Online Resource 1**

INTEGRATIVE GENETIC, EPIGENETIC AND PATHOLOGICAL ANALYSIS OF PARAGANGLIOMA REVEALS COMPLEX DYSREGULATION OF NOTCH SIGNALING

Alessandro Cama, Fabio Verginelli, Lavinia Vittoria Lotti Francesco Napolitano, Annalisa Morgano, Andria D'Orazio, Michele Vacca, Silvia Perconti, Felice Pepe, Federico Romani, Francesca Vitullo, Filippo di Lella, Rosa Visone, Massimo Mannelli, Hartmut P.H. Neumann, Giancarlo Raiconi, Carlo Paties, Antonio Moschetta, Roberto Tagliaferri, Angelo Veronese, Mario Sanna, Renato Mariani-Costantini.

**Corresponding author:** Professor Renato Mariani-Costantini, MD, Unit of General Pathology, Aging Research Center (Ce.S.I.), *G. d'Annunzio* University Foundation, *Via Colle dell'Ara*, 66100 Chieti, Italy. Tel. +39-0871541496, e-mail: rmc@unich.it.

The file contains the following Supplementary Tables: synopsis of the 28 prospectively-sampled paraganglioma cases investigated in the study **(Supplementary Table 1);** table detailing case data, *SDH* mutations and SDHB immunohistochemistry **(Supplementary Table 2);** synopsis of the NOTCH1-related immunohistochemical and pathological variables **(Supplementary Table 3);** synopsis of the data concerning the18 Jacobson's nerve samples that served as controls for RNA and miRNA studies (**Supplementary Table 4);** list of the oligonucleotide sequences used for cloning, qRT-PCR and site direct mutagenesis **(Supplementary Table 5**); list of miRNA mimics used in transfection experiments **(Supplementary Table 6**); list of the genes showing highest concordance for tumor-associated CNVs **(Supplementary Table 7**); list of the most significant functional-related gene groups identified using DAVID **(Supplementary Table 8**); table detailing the fractions of the mutation carriers for the *SDHB*, *SDHC*, *SDHD* and *SDHAF2* genes and of the cases with tumor associated loss of SDHB in the subsets of head and neck paragangliomas analyzed for *SDH* germline mutations (**Supplementary Table 9)**; table showing the individual characteristics, the fraction with *SDH* mutations and the fraction with loss of SDHB immunostaining in the PGL subsets with different clinical presentation **(Supplementary Table 10)**; table showing the mean NOTCH1 and JAG2 immunostaining intensities in chief cells, sustentacular cells and endothelial cells of the paraganglioma subsets defined according to *SDH* mutation status and SDHB immunohistochemistry (**Supplementary Table 11).**

**Supplementary Table 1 - Synopsis of the 28 prospectively-sampled paraganglioma cases (29 individual tumors) investigated in the present study.** The table details the type(s) of donated sample(s) and the studies performed in relation to the individual and clinico-pathological data. Acronyms include case number, followed by abbreviations referring to paraganglioma (P) localization: PTJ, tympano-jugular; PT, tympanic; PC, carotid body; PV, vagal. #: 5PC and 5PV are distinct paragangliomas originating in the same patient, respectively in the right carotid body and along the left vagus nerve. Bold characters in "Disease localization(s)" indicate the primary tumor analyzed in the current study. na: Fisch classification not applicable. Acronyms for study procedures are detailed in the printed text.

| Acronym | Gender | Age (yrs) at surgery | Disease localization(s) | Family history | Modified Fisch class* | Studies |
|---|---|---|---|---|---|---|
| 1PTJ | F | 25 | mediastinal; left vagal, **left tympano-jugular** (recurrence); left carotid body | yes | C2 | CNV, qPCR, miRNA expression profiling, qRT-PCR, IHC |
| 2PTJ | M | 59 | **right tympano-jugular** | no | C2 | CNV, qPCR, miRNA expression profiling, qRT-PCR, IHC, EM, Cryo-IEM, IF |
| 3PT | F | 73 | **right tympanic** | no | A2 | CNV, qPCR, miRNA expression profiling, qRT-PCR, IHC |
| 4PTJ | M | 42 | **right tympano-jugular** | no | C3 | CNV, qPCR, miRNA expression profiling, qRT-PCR, IHC, EM, Cryo-IEM, IF |
| 5PC# | F | 52 | **right carotid body**; left tympano-jugular; left vagal | no | na | CNV, qPCR, miRNA expression profiling, qRT-PCR, IHC, EM, Cryo-IEM, IF |
| 5PV# | F | | right carotid body; left tympano-jugular; **left vagal** | no | na | CNV, qPCR, miRNA expression profiling, qRT-PCR |
| 6PTJ | F | 52 | **right tympano-jugular** | no | C2 | CNV, qPCR, miRNA expression profiling, qRT-PCR, IHC, EM, Cryo-IEM, IF |
| 7PC | F | 42 | **right carotid body**; left tympano-jugular | no | na | CNV, qPCR, miRNA expression profiling, qRT-PCR, IHC, EM, Cryo-IEM, IF |
| 8PTJ | M | 31 | **right tympano-jugular** | no | C3 | CNV, qPCR, IHC, EM, Cryo-IEM, IF |
| 11PT | M | 57 | **left tympanic** | no | B3 | CNV, qPCR, EM, Cryo-IEM, IF |
| 12PTJ | F | 74 | **left tympano-jugular** | no | A2 | CNV, qPCR, EM, Cryo-IEM, IF |
| 13PTJ | M | 58 | **right tympano-jugular** | no | C3 | CNV, qPCR, IHC, EM, CIEM, IF |
| 14PT | M | 43 | **left tympanic** | no | A | CNV, qPCR, miRNA expression profiling, qRT-PCR |
| 19PT | | 74 | **right tympanic** | no | B3 | CNV, qPCR |
| 20PT | M | 51 | **right tympanic** | no | B3 | CNV, qPCR, IHC, EM, Cryo-IEM, IF |
| 21PTJ | M | 34 | **right tympano-jugular** | no | C3 | CNV, qPCR, miRNA expression profiling, qRT-PCR, IHC |
| 32PT | M | 56 | **right tympanic** | no | A2 | CNV, qPCR, miRNA expression profiling, qRT-PCR |
| 33PT | F | 52 | **right tympanic** | no | B3 | CNV, qPCR, miRNA expression profiling, qRT-PCR |
| 34PTJ | F | 25 | **left tympano-jugular** (recurrence); metastases in 4/17 regional lymph nodes | no | C2 | CNV, qPCR, IHC |
| 36PT | F | 60 | **right tympanic** | no | B3 | CNV, qPCR, IHC |
| 37PTJ | F | 63 | **right tympano-jugular** | no | C1 | CNV, qPCR, miRNA expression profiling, IHC, qRT-PCR |
| 43PTJ | F | 68 | **right tympano-jugular** | no | C1 | CNV, qPCR, IHC, qRT-PCR |
| 44PT | F | 51 | **right tympanic** | no | B3 | CNV, IHC, qRT-PCR |
| 45PTJ | M | 35 | **left tympano-jugular** | no | C3 | CNV, qPCR, IHC |
| 54PTJ | M | 34 | right carotid body; **left tympano-jugular** | no | C2 | qPCR, IHC, IF, EM, Cryo-IEM |
| 57PTJ | F | 41 | **left tympano-jugular** | no | C2 | qPCR, IHC, IF, EM, Cryo-IEM |
| 58PTJ | M | 43 | **right tympano-jugular** | no | C2 | qPCR, IHC, IF, EM, Cryo-IEM |
| 59PTJ | F | 53 | **left tympano-jugular** | no | C1 | qPCR, IHC, IF, EM, Cryo-IEM |
| 64PTJ | M | 33 | **right tympano-jugular** | no | C1 | qPCR, qRT-PCR, IHC, IF, EM, Cryo-IEM, cell culture |

*Shin SH, Sivalingam S, De Donato G, Falcioni M, Piazza P, Sanna M. Vertebral artery involvement by tympanojugular paragangliomas: management and outcomes with a proposed addition to the Fisch classification. Audiol Neurootol. 2012;17:92-104.

**Supplementary Table 2 - Case data, *SDH* mutations and SDHB immunohistochemistry.** The table details 47 tumors from 46 patients, selected for quality/quantity of the paraffin-embedded material and corresponding to those in Supplementary Table 3. Prospective case acronyms include case number followed by abbreviations for paraganglioma (P) localization: PTJ, tympano-jugular; PT, tympanic; PC, carotid body; PV, vagal. Retrospective (R) cases are indicated by the tumor acronym, followed by the last two digits of the year, R for retrospective and case number. PC-06/R15A and PV-06/R15b are independent tumors from one case. na = not available.

| Case acronym | Individual and clinical data | | | | | Mutational analysis | | | | SDHB IHC |
|---|---|---|---|---|---|---|---|---|---|---|
| | **Age** | **Gender** | **Family history** | **Tumor localization(s)** | **Multiple/ Recurrent** | **Genes** | **Mutation type** | **Mutation nucleotide** | **Mutation protein** | |
| **1PTJ** | 25 | F | yes | PTJ(lt), PV(lt), PC(bil) | yes | undetected | | | | negative |
| **2PTJ** | 59 | M | no | PTJ(rt) | no | undetected | | | | positive |
| **3PT** | 73 | F | no | PT(rt) | no | na | | | | positive |
| **4PTJ** | 42 | M | no | PTJ(rt) | yes | undetected | | | | positive |
| **5PC** | 52 | F | no | PTJ(lt), PV(lt), PC(rt) | yes | SDHD | FS | c.445_448dupATCT | Truncation | negative |
| **6PTJ** | 52 | F | no | PTJ(rt) | no | na | | | | positive |
| **7PC** | 42 | F | no | PTJ(lt), PC(rt) | yes | undetected | | | | negative |
| **8PTJ** | 31 | M | no | PTJ(rt) | no | SDHB | Large deletion/ Rearrangement | na | na | negative |
| **13PTJ** | 58 | M | no | PTJ(rt) | no | undetected | | | | positive |
| **20PT** | 51 | M | no | PT(rt) | no | na | | | | positive |
| **21PTJ** | 34 | M | no | PTJ(rt) | no | SDHB | FS | c.575dupT | p.C192L fsX1 | negative |
| **34PTJ** | 25 | F | no | PTJ(lt) | yes (metastatic) | SDHB | MS | c.912G>C | G260R | negative |
| **36PT** | 60 | F | no | PT(rt) | no | na | | | | positive |
| **37PTJ** | 63 | F | no | PTJ(rt) | no | na | | | | positive |
| **43PTJ** | 52 | F | no | PTJ(rt) | no | na | | | | positive |
| **44PT** | 51 | F | no | PT(rt) | no | na | | | | negative |
| **45PTJ** | 39 | M | no | PTJ(lt) | no | undetected | | | | positive |
| **54PTJ** | 34 | M | no | PC(rt), PTJ(lt) | yes | na | | | | negative |
| **57PTJ** | 41 | F | no | PTJ(lt) | no | undetected | | | | positive |
| **58PTJ** | 43 | M | no | PTJ(rt) | yes | SDHC | Splice site alteration | c.241+1G>A | Truncation | positive |
| **59PTJ** | 53 | F | no | PTJ(lt) | no | na | | | | positive |
| **64PTJ** | 33 | M | no | PTJ(rt) | no | na | | | | positive |

*Continued*

| Case acronym | Individual and clinical data | | | | | SDHx genes mutational analysis | | | | SDHB IHC |
|---|---|---|---|---|---|---|---|---|---|---|
| | Age | Gender | Family history | Tumor localization(s) | Multiple/ Recurrent | Genes | Mutation type | Mutation nucleotide | Mutation protein | |
| **PTJ-02/R1** | 44 | F | no | PTJ(rt) | no | undetected | | | | positive |
| **PTJ-03/R2** | 47 | F | no | PTJ(lt) | no | undetected | | | | positive |
| **PC-03/R3** | 31 | M | no | PTJ(rt), PC(bil) | yes | SDHD | Large deletion/ Rearrangement | 5'UTR_ exon1_exon 2 del | Truncation | negative |
| **PTJ-04/R4** | 27 | M | no | PTJ(lt) | no | undetected | | | | positive |
| **PTJ-04/R5** | 31 | M | no | PTJ(rt) | no | undetected | | | | negative |
| **PTJ-04/R6** | 24 | M | no | PTJ(rt) | no | SDHAF2 | MS | c.232G>C | p.G78A | negative |
| **PTJ-04/R7** | 67 | F | no | PTJ(rt) | no | undetected | | | | positive |
| **PTJ-04/R8** | 63 | F | no | PTJ(lt) | no | undetected | | | | positive |
| **PTJ-04/R9** | 53 | F | no | PTJ(lt) | no | undetected | | | | positive |
| **PTJ-05/R10** | 32 | F | no | PTJ(lt) | no | undetected | | | | negative |
| **PTJ-05/R11** | 55 | F | no | PTJ(rt) | no | undetected | | | | positive |
| **PTJ-05/R12** | 42 | F | no | PTJ(lt) | no | undetected | | | | negative |
| **PTJ-05/R13** | 44 | F | no | PTJ(lt) | no | undetected | | | | negative |
| **PC-06/R14** | 49 | F | no | PTJ(lt), PC(bil) | yes | SDHD | MS | c.242C>T | p.P81L | negative |
| **PC-06/R15a** / **PV-06/R15b** | 59 | F | na | PV(lt), PC(lt) | yes | SDHD | MS | c.242C>T | p.P81L | negative |
| **PTJ-06/R16** | 64 | F | no | PTJ(lt) | no | undetected | | | | positive |
| **PTJ-06/R17** | 55 | M | no | PTJ(lt) | no | na | | | | negative |
| **PV-06/R18** | 27 | F | no | PV(rt) | no | SDHB | NS | c.275G>A | p.W47X | negative |
| **PTJ-06/R19** | 55 | M | no | PTJ(lt), PV(lt) | yes | SDHB | MS | c.232G>C | p.G78A | negative |
| **PTJ-06/R20** | 40 | F | no | PTJ(rt) | no | SDHB | Splice site alteration | c.557+1G>A | Truncation | negative |
| **PTJ-08/R21** | 45 | M | no | PTJ(lt) | no | SDHD | MS | c.341A>G | p.Y114C | positive |
| **PTJ-10/R22** | 37 | M | no | PTJ(lt) | no | undetected | | | | na |
| **PTJ-11/R23** | 45 | F | no | PTJ(rt) | no | undetected | | | | positive |
| **PV-11/R24** | 49 | F | no | PV(bil) | yes | SDHD | FS | c.445_448dupATCT | Truncation | negative |

*Supplementary Table 2*

**Supplementary Table 3 - Synopsis of the NOTCH1-related immunohistochemical and pathological variables.** The 47 individual paraffin-embedded tumors from 46 patients correspond to those detailed in Supplementary Table 2, which provides complementary information. The tumors include 22 of the prospectively-collected cases in supplementary Table 1 (with known CNV status at *NOTCH1* and *JAG2*; gain = 3 or more copies, no change = 2 copies), plus 24 archival cases (with 25 tumors, PC-06/R15a and PV-06/R15b are independent tumors from the same patient). Immunostaining results were evaluated both in terms of % positive cells (in brackets), counted in 4 high magnification fields (400x, each field estimated to contain 250-400 cells), and of intensity, scored on a semiquantitative scale (0 = no staining; 1 = weak but definitely positive staining; 2 = moderate staining; 3 = strong staining). Immunostaining for NOTCH1, JAG2, S100 and BCL2 is detailed for the 3 main PGL cell types (chief cells: CC; sustentacular cells: SC; endothelial cells: EC); immunostaining for synaptophysin (SYN) refers to chief and sustentacular cells (CC and SC) together, as these cell types were similarly and strongly labelled (endothelia were negative), immunostaining for vimentin (VIM) to all cell types (chief, sustentacular, endothelial). Ki67 is evaluated in terms of % of positively-stained nuclei (chief and/or sustentacular cells), counted in 4 high magnification fields. Other routinely assessed clinicopathological variables include presence (yes) or absence (no) of vascular invasion, bone infiltration and atypia. Prospective case acronyms include case number followed by abbreviations referring to paraganglioma (P) localization: PTJ, tympano-jugular; PT, tympanic; PC, carotid body; PV, vagal. Retrospective (R) cases are indicated by tumor acronym, followed by the last two digits of the year, R and case number. na = not available. Asterisks in the NOTCH1 and JAG2 CNV columns indicate 3 cases for which CNVs were assessed by qPCR analysis only.

| Case acronym | NOTCH1 | | | | JAG2 | | | | SYN intensity (%) | CGA intensity (%) | S100 | | | VIM intensity(%) | Ki67 positive cells (%) | BCL2 | | | Vascular invasion | Bone infiltration | Atypia |
|---|---|---|---|---|---|---|---|---|---|---|---|---|---|---|---|---|---|---|---|---|---|
| | CNV | IHC intensity (%) CC | SC | EC | CNV | IHC intensity (%) CC | SC | EC | | | IHC intensity (%) CC | SC | EC | | | IHC intensity (%) CC | SC | EC | | | |
| **1PTJ** | 3 | 1 (100) | 2 (100) | 2 (100) | 3 | 1 (100) | 3 (100) | 0 (100) | 3 (100) | 1(70) 2(30) | 0 (100) | 3 (100) | 0 (100) | 3 (90) | 1 | 0 (100) | 2 (100) | 1 (20) | no | no | yes |
| **2PTJ** | 2 | 2 (100) | 2 (100) | 3 (100) | 2 | 1 (100) | 1 (100) | 0 (100) | 2 (100) | 0 (100) | 2 (20) | 3 (5) | 0 (100) | 2 (100) | 1 | 0 (100) | 1 (100) | 1 (100) | no | no | no |
| **3PT** | 3 | 2 (100) | 2 (100) | 3 (100) | 4 | 1 (100) | 1 (100) | 0 (100) | 3 (100) | na | 1 (100) | 3 (100) | 0 (100) | 3 (100) | 1 | 0 (100) | 1 (100) | 0 (100) | no | yes | no |
| **4PTJ** | 3 | 1 (100) | 1 (100) | 2 (100) | 3 | 1 (100) | 1 (100) | 0 (100) | 2 (100) | 3 (100) | na | na | 0 (100) | 3 (80) | 1 | 0 (100) | 1 (100) | 2 (100) | no | yes | yes |
| **5PC** | 3 | 2 (100) | 2 (100) | 3 (100) | 3 | 1 (100) | 2 (100) | 0 (100) | 3 (100) | 1 (80) 3(20) | 0 (100) | 3 (100) | 0 (100) | 3 (100) | 7 | 0 (100) | 1 (100) | 1 (100) | no | no | yes |
| **6PTJ** | 3 | 2 (100) | 3 (100) | 2 (100) | 3 | 1 (100) | 3 (100) | 0 (100) | 3 (100) | 1 (60) 3(40) | 0 (100) | 3 (100) | 0 (100) | 3 (100) | 1 | 0 (100) | 2 (100) | 1 (100) | no | no | no |
| **7PC** | 3 | 1 (100) | 1 (100) | 2 (100) | 3 | 1 (100) | 2 (100) | 0 (100) | 3 (100) | 1 (60) 2(40) | 0 (100) | 3 (100) | 0 (100) | 3 (100) | 1 | 0 (100) | 1 (100) | 1 (100) | no | no | no |
| **8PTJ** | 3 | 2 (100) | 2 (100) | 3 (100) | 2 | 0 (100) | 0 (100) | 0 (100) | 3 (100) | 1 (20) 2(80) | 3 (100) | 3 (100) | 0 (100) | 3 (100) | 1 | 0 (100) | 1 (100) | 1 (100) | no | yes | yes |
| **13PTJ** | 3 | 2 (100) | 2 (100) | 3 (100) | 2 | 1 (100) | 1 (100) | 0 (100) | 3 (100) | 1 (50) 2(10) | 0 (100) | 3 (100) | 0 (100) | 3 (100) | 1 | 1 (100) | 2 (100) | 1 (100) | no | no | no |
| **20PT** | 2 | 3 (100) | 3 (100) | 3 (100) | 3 | 1 (100) | 1 (100) | 0 (100) | 3 (100) | na | 0 (100) | 3 (100) | 0 (100) | 3 (100) | 3 | 0 (100) | 2 (100) | 0 (100) | no | no | no |
| **21PTJ** | 3 | 1 (100) | 1 (100) | 2 (100) | 3 | 1 (100) | 1 (100) | 0 (100) | 3 (100) | 1 (60) 2(40) | 0 (100) | 3 (100) | 0 (100) | 1 (30) | 3 | 0 (100) | 1 (100) | 1 (100) | yes | no | no |
| **34PTJ** | 3 | 1 (100) | 1 (100) | 2 (100) | 3 | 0 (100) | 0 (100) | 0 (100) | 3 (100) | 1 (60) 3(40) | 0 (100) | 3 (100) | 0 (100) | 3 (100) | 2 | 0 (100) | 1 (100) | 1 (100) | no | yes | no |
| **36PT** | 3 | 2 (100) | 3 (100) | 3 (100) | 3 | 1 (100) | 3 (5) | 0 (100) | 3 (100) | 1 (90) 3(10) | 0 (100) | 3 (100) | 0 (100) | 2 (100) | 1 | 0 (100) | 1 (100) | 1 (100) | no | yes | yes |
| **37PTJ** | 3 | 1 (100) | 1 (100) | 2 (100) | 3 | 1 (100) | 2 (100) | 0 (100) | 3 (100) | 1 (80) 3(20) | 0 (100) | 3 (100) | 0 (100) | 3 (100) | 1 | 0 (100) | 2 (100) | 0 (100) | no | no | no |
| **43PTJ** | 3 | 3 (100) | 3 (100) | 3 (100) | 2 | 1 (100) | 2 (100) | 0 (100) | 3 (100) | 1 (20) 3(10) | 0 (100) | 3 (100) | 0 (100) | 0 (100) | 1 | 0 (100) | 2 (100) | 1 (40) | no | yes | no |
| **44PT** | 2 | 2 (100) | 2 (100) | 3 (100) | 2 | 2 (100) | 2 (100) | 0 (100) | 3 (100) | 1 (20) | 2 (100) | 3 (100) | 0 (100) | 3 (100) | 1 | 1 (70) 2 (30) | 1 (70) 2 (30) | 1 (50) | no | yes | no |

*Continued*

| Case acronym | NOTCH1 | | | | JAG2 | | | | SYN intensity (%) | CGA intensity (%) | S100 | | | VIM intensity(%) | Ki67 positive cells (%) | BCL2 | | | Vascular invasion | Bone infiltration | Atypia |
|---|---|---|---|---|---|---|---|---|---|---|---|---|---|---|---|---|---|---|---|---|---|
| | CNV | IHC intensity (%) | | | CNV | IHC intensity (%) | | | | | IHC intensity (%) | | | | | IHCintensity (%) | | | | | |
| | | CC | SC | EC | | CC | SC | EC | | | CC | SC | EC | | | CC | SC | EC | | | |
| **45PTJ** | 3 | 2 (100) | 2 (100) | 3 (100) | 2 | na | na | na | na | 3 (10) | 0 (100) | 3 (100) | 0 (100) | 3 (100) | 1 | 2 (100) | 2 (100) | 0 (100) | no | yes | no |
| **54PTJ** | 2* | 2 (100) | 2 (100) | 3 (100) | 2* | na | na | na | 3 (100) | na | 1 (100) | 3 (100) | 0 (100) | 3 (100) | 1 | na | na | na | no | yes | no |
| **57PTJ** | na | 2 (100) | 2 (100) | 3 (100) | na | 1 (100) | 1 (100) | 0 (100) | 3 (100) | 3 (100) | 0 (100) | 3 (100) | 0 (100) | 3 (100) | 2 | 0 (100) | 1 (100) | 1 (100) | no | no | no |
| **58PTJ** | 2* | 2 (100) | 2 (100) | 3 (100) | 2* | 1 (100) | 1 (100) | 0 (100) | 3 (100) | 1 (80) 3 (20) | 1 (100) | 3 (100) | 0 (100) | 1 (100) | 1 | 0 (100) | 1 (100) | 1 (100) | yes | no | yes |
| **59PTJ** | 2* | 2 (100) | 2 (100) | 3 (100) | 2* | 1 (100) | 1 (100) | 0 (100) | 3 (100) | 2 (70) 3 (30) | 3 (100) | 3 (100) | 0 (100) | 0 (100) | < 1 | 0 (100) | 0 (100) | 1 (100) | no | no | no |
| **64PTJ** | 2* | 2 (100) | 2 (100) | 3 (100) | 3* | 1 (100) | 1 (100) | 0 (100) | na | 1 (50) 2 (40) | 1 (50) | 3 (100) | 0 (100) | na | na | na | na | na | no | na | na |
| **PTJ-02/R1** | na | 1 (100) | 1 (100) | 2 (100) | na | 0 (100) | 1 (100) | 0 (100) | 2 (100) | 1 (80) 2 (20) | 0 (100) | 3 (100) | 0 (100) | 3 (100) | 1 | 0 (100) | 1 (100) | 1 (100) | no | no | no |
| **PTJ-03/R2** | na | 2 (100) | 2 (100) | 3 (100) | na | 1 (100) | 1 (100) | 0 (100) | 2 (100) | 2 (40) | 1 (100) | 2 (100) | 0 (100) | 2 (100) | < 1 | 0 (100) | 2 (100) | 1 (100) | no | yes | no |
| **PC-03/R3** | na | 2 (100) | 2 (100) | 3 (100) | na | 1 (100) | 1 (100) | 0 (100) | 3 (100) | 1 (80) 2 (20) | 0 (100) | 3 (100) | 0 (100) | 3 (60)1 (40) | < 1 | 0 (100) | 1 (50) | 0 (100) | no | no | yes |
| **PTJ-04/R4** | na | 2 (100) | 2 (100) | 3 (100) | na | 1 (100) | 1 (100) | 0 (100) | 3 (100) | 3 (60) | 0 (100) | 3 (100) | 0 (100) | 3 (100) | < 1 | 0 (100) | 1 (100) | 0 (100) | no | yes | no |
| **PTJ-04/R5** | na | 1 (100) | 2 (100) | 2 (100) | na | 1 (100) | 1 (100) | 0 (100) | 3 (100) | 1 (50) 2 (40) | 1 (100) | 3 (100) | 0 (100) | 3 (100) | 1 | 0 (100) | 0 (100) | 0 (100) | no | no | yes |
| **PTJ-04/R6** | na | 2 (100) | 3 (100) | 3 (100) | na | 1 (100) | 1 (100) | 0 (100) | 2 (100) | 1 (100) | 0 (100) | 3 (100) | 0 (100) | 3 (100) | na | 0 (100) | 1 (100) | 1 (100) | no | no | yes |
| **PTJ-04/R7** | na | 2 (100) | 2 (100) | 3 (100) | na | 2 (100) | 2 (100) | 0 (100) | 3 (100) | 3 (100) | 2 (100) | 3 (100) | 0 (100) | 2 (100) | 1 | 0 (100) | 1 (100) | 1 (100) | no | no | no |
| **PTJ-04/R8** | na | 2 (100) | 2 (100) | 3 (100) | na | 1 (100) | 1 (100) | 0 (100) | 2 (100) | 2 (80) 3 (20) | 0 (100) | 3 (100) | 0 (100) | 0 (100) | < 1 | 3 (100) | 3 (100) | 1 (40) | no | yes | no |
| **PTJ-04/R9** | na | 2 (100) | 3 (100) | 3 (100) | na | 1 (100) | 1 (100) | 0 (100) | 3 (100) | 1 (70) 2 (30) | 0 (100) | 3 (100) | 0 (100) | 2 (100) | < 1 | 0 (100) | 1 (100) | 0 (100) | no | no | yes |
| **PTJ-05/R10** | na | 2 (100) | 2 (100) | 3 (100) | na | 1 (100) | 1 (100) | 0 (100) | 3 (100) | 1 (60) 2 (40) | 0 (100) | 3 (100) | 0 (100) | 3 (100) | < 1 | 0 (100) | 1 (10) | 0 (100) | no | yes | yes |
| **PTJ-05/R11** | na | 2 (100) | 2 (100) | 3 (100) | na | 1 (100) | 1 (100) | 0 (100) | 3 (100) | 1 (25) 2 (5) | 0 (100) | 3 (100) | 0 (100) | 2 (100) | < 1 | 0 (100) | 1 (50) | 1 (100) | no | yes | no |
| **PTJ-05/R12** | na | 2 (100) | 2 (100) | 3 (100) | na | 1 (100) | 1 (100) | 0 (100) | 2 (100) | 3 (30) | 0 (100) | 3 (100) | 0 (100) | 3 (100) | 2 | 0 (100) | 0 (100) | 1 (40) | no | yes | no |
| **PTJ-05/R13** | na | 2 (100) | 2 (100) | 3 (100) | na | 1 (100) | 1 (100) | 0 (100) | 3 (100) | 2 (100) | 1 (100) | 3 (100) | 0 (100) | 3 (100) | < 1 | 0 (100) | 1 (100) | 0 (100) | no | no | no |
| **PC-06/R14** | na | 2 (100) | 2 (100) | 3 (100) | na | 1 (100) | 1 (100) | 0 (100) | 2 (100) | 3 (100) | 1 (100) | 2 (100) | 0 (100) | 2 (100) | < 1 | 0 (100) | 1 (100) | 2 (100) | no | no | yes |
| **PC-06/R15a** | na | 2 (100) | 2 (100) | 3 (100) | na | 0 (100) | 0 (100) | 0 (100) | 3 (100) | 2 (40) 3 (60) | 0 (100) | 3 (100) | 0 (100) | 3 (100) | < 1 | 0 (100) | 1 (100) | 1 (100) | no | no | yes |
| **PV-06/R15b** | na | 2 (100) | 2 (100) | 3 (100) | na | 1 (100) | 1 (100) | 0 (100) | 3 (100) | 1 (1) | 2 (100) | 3 (100) | 0 (100) | 3 (100) | < 1 | 0 (100) | 1 (100) | 1 (100) | no | no | yes |
| **PTJ-06/R16** | na | 2 (100) | 2 (100) | 3 (100) | na | 1 (100) | 1 (100) | 0 (100) | 3 (100) | 1 (40) 2 (20) | 0 (100) | 3 (100) | 0 (100) | 3 (100) | 1 | 0 (100) | 1 (100) | 1 (100) | no | no | no |
| **PTJ-06/R17** | na | 2 (100) | 2 (100) | 3 (100) | na | 1 (100) | 1 (100) | 0 (100) | 3 (100) | 2 (80) 3 (20) | 0 (100) | 3 (100) | 0 (100) | 3 (100) | < 1 | 0 (100) | 0 (100) | 0 (100) | no | yes | no |
| **PV-06/R18** | na | 2 (100) | 3 (100) | 3 (100) | na | 1 (100) | 2 (100) | 0 (100) | 3 (100) | 1 (20) 2 (60) | 0 (100) | 3 (100) | 0 (100) | 3 (100) | 1 | 0 (100) | 1 (100) | 1 (100) | no | no | yes |

*Supplementary Table 3 continued*

| Case acronym | NOTCH1 | | | | JAG2 | | | | SYN intensity (%) | CGA intensity (%) | S100 | | | VIM intensity(%) | Ki67 positive cells (%) | BCL2 | | | Vascular invasion | Bone infiltration | Atypia |
|---|---|---|---|---|---|---|---|---|---|---|---|---|---|---|---|---|---|---|---|---|---|
| | CNV | IHC intensity (%) | | | CNV | IHC intensity (%) | | | | | IHC intensity (%) | | | | | IHCintensity (%) | | | | | |
| | | CC | SC | EC | | CC | SC | EC | | | CC | SC | EC | | | CC | SC | EC | | | |
| **PTJ-06/R19** | na | 2 (100) | 2 (100) | 3 (100) | na | 1 (100) | 1 (100) | 0 (100) | 2 (100) | 3 (100) | 0 (100) | 3 (100) | 0 (100) | 1 (70) 2 (30) | 1 | 0 (100) | 1 (100) | 1 (100) | no | yes | yes |
| **PTJ-06/R20** | na | 2 (100) | 2 (100) | 3 (100) | na | 1 (100) | 1 (100) | 0 (100) | 3 (100) | 3 (100) | 0 (100) | 3 (100) | 0 (100) | 3 (100) | 1 | 0 (100) | 1 (100) | 1 (100) | no | yes | yes |
| **PTJ-08/R21** | na | 1 (100) | 1 (100) | 2 (100) | na | 1 (100) | 1 (100) | 0 (100) | 3 (100) | na | 0 (100) | 3 (10) | 0 (100) | 2 (100) | 1 | 0 (100) | 1 (100) | 2 (100) | no | yes | no |
| **PTJ-10/R22** | na | 2 (100) | 2 (100) | 3 (100) | na | 1 (100) | 1 (100) | 0 (100) | 3 (100) | 1 (70) 2 (30) | 0 (100) | 3 (100) | 0 (100) | 3 (80) | 2 | 0 (100) | 1 (100) | 1 (100) | no | yes | yes |
| **PTJ-11/R23** | na | 2 (100) | 2 (100) | 3 (100) | na | 1 (100) | 1 (100) | 0 (100) | 3 (100) | 1 (30) 2 (10) | 3 (40) | 3 (100) | 0 (100) | 3 (90) | 1 | 0 (100) | 1 (100) | 0 (100) | no | yes | no |
| **PV-11/R24** | na | 2 (100) | 2 (100) | 3 (100) | na | 1 (100) | 1 (100) | 0 (100) | 3 (100) | 3 (100) | 0 (100) | 3 (100) | 0 (100) | 2 (100) | < 1 | 0 (100) | 1 (60) | 2 (60) | no | no | no |

*Supplementary Table 3*

**Supplementary Table 4 - Synopsis of the 18 control Jacobson's nerve samples.** The Table reports individual and clinico-pathological data, including donor age, gender, disease for which surgery requiring nerve removal was performed, sample type(s) and use(s) in the present study. Due to minute sample sizes and very low concentrations, most RNAs from Jacobson's nerves (JNs) were pooled in sets, each including 2-5 patients (depending on RNA content). Each individual nerve included in pools contributed the same quantity of RNA, (na = not available, other abbreviations as detailed in the printed text).

| Acronym | Gender | Age | Disease | Sample | Studies |
|---|---|---|---|---|---|
| **JN5** | F | 39 | vestibular schwannoma | normal nerve, blood | RNA pool (CTRL6), miRNA expression profiling |
| **JN6** | M | 40 | hemangioma | normal nerve, blood | RNA pool (CTRL6), miRNA expression profiling |
| **JN8** | M | 41 | vestibular schwannoma | normal nerve, blood | RNA pool (CTRL8), miRNA expression profiling |
| **JN9** | F | 70 | vestibular schwannoma | normal nerve, blood | RNA pool (CTRL8), miRNA expression profiling |
| **JN10** | F | 59 | vestibular schwannoma | normal nerve, blood | RNA pool (CTRL10), miRNA expression profiling |
| **JN12** | F | 61 | vestibular schwannoma | normal nerve, blood | RNA pool (CTRL8), miRNA expression profiling |
| **JN14** | M | 40 | vestibular schwannoma | normal nerve, blood | RNA pool (CTRL10), miRNA expression profiling |
| **JN23** | F | 55 | vestibular schwannoma | normal nerve, blood | miRNA expression profiling, qRT-PCR |
| **JN25** | M | 36 | vestibular schwannoma | normal nerve, blood | RNA pool (CTRL21), miRNA expression profiling |
| **JN26** | F | 59 | vestibular schwannoma | normal nerve, blood | RNA pool (CTRL21), miRNA expression profiling |
| **JN27** | F | 49 | vestibular schwannoma | normal nerve, blood | RNA pool (CTRL21), miRNA expression profiling |
| **JN50** | M | 47 | vestibular schwannoma | normal nerve, blood | RNA pool (CTRL21), miRNA expression profiling |
| **JN51** | F | 41 | vestibular schwannoma | normal nerve, blood | RNA pool (CTRL21), miRNA expression profiling |
| **JN44** | F | na | vestibular schwannoma | normal nerve, blood | qRT-PCR |
| **JN46** | M | 65 | vestibular schwannoma | normal nerve, blood | qRT-PCR |
| **JN47** | F | na | vestibular schwannoma | normal nerve, blood | qRT-PCR |
| **JN48** | M | 38 | vestibular schwannoma | normal nerve, blood | qRT-PCR |
| **JN49** | F | na | vestibular schwannoma | normal nerve, blood | qRT-PCR |

**Supplementary Table 5 -** Oligonucleotide sequences used for cloning, qRT-PCR and site direct mutagenesis.

| | *Gene* | *Name* | *UPL probe** | *Sequence* |
|---|---|---|---|---|
| **Cloning** | NOTCH1 | NOTCH1_7745F_X | - | CAACTCGAGCGACCAGAGGAGCCTTTTTA |
| | NOTCH1 | NOTCH1_ 8664R_X | - | CAACTCGAGTGTGTTGCTGGAGCATCTTC |
| **qRT-PCRs primer** | NOTCH1 | u85_NOTCH1_F | #85 | CGCACAAGGTGTCTTCCAG |
| | NOTCH1 | u85_NOTCH1_R | #85 | AGGATCAGTGGCGTCGTG |
| | JAG2 | u17_JAG2_F | #17 | TGGGACTGGGACAACGATAC |
| | JAG2 | u17_JAG2_R | #17 | ATGCGACACTCGCTCGAT |
| | HES1 | u60_HES1_F | #60 | GAAGCACCTCCGGAACCT |
| | HES1 | u60_HES1_R | #60 | GTCACCTCGTTCATGCACTC |
| | HES5 | u70_HES5_F | #70 | TCAGCTACCTGAAGCACAGC |
| | HES5 | u70_HES5_R | #70 | TAGTCCTGGTGCAGGCTCTT |
| | hsa-miR-34b-5p | 34b_F | #21 | GCGGCGGTAGGCAGTGTCATT |
| | hsa-miR-34c-5p | 34c_F | #21 | GCGGCGGAGGCAGTGTAGTTAG |
| | hsa-miR-200a-3p | 200a_F | #21 | GCGGCGGTAACACTGTCTGGTA |
| | hsa-miR-200b-3p | 200b_F | #21 | GCGGCGGTAATACTGCCTGGT |
| | hsa-miR-200c-3p | 200c_F | #21 | GCGGCGGTAATACTGCCGGGTA |
| | hsa-miR-129-3p | 129_F | #21 | GCGGCGGAAGCCCTTACCCCAA |
| | hsa-miR-431-5p | 431_F | #21 | GCGGCGGTGTCTTGCAGGCCG |
| | hsa-miR-127-5p | 127_F | #21 | GCGGCGGCTGAAGCTCAGAGG |
| | hsa-miR-433 | 433_F | #21 | GCGGCGGATCATGATGGGCTC |
| | hsa-miR-483-3p | 483_F | #21 | GCGGCGGTCACTCCTCTCCTC |
| | RNU6B | U6B_F | #21 | GCGGCGGCGCAAGGATGACACG |
| | Universal R | UniR | #21 | GTGCAGGGTCCGAGGT |
| | hACTB | | | Applied assay Cat. # 4331182 |
| **Stem loop RT primers** | hsa-miR-34b-5p | RT_34b-5p | #21 | GTTGGCTCTGGTGCAGGGTCCGAGGTATTCGCACCAGAGCCAACCAATCAG |
| | hsa-miR-34c-5p | RT_34c-5p | #21 | GTTGGCTCTGGTGCAGGGTCCGAGGTATTCGCACCAGAGCCAACGCAATC |
| | hsa-miR-200a-3p | RT_200a-3p | #21 | GTTGGCTCTGGTGCAGGGTCCGAGGTATTCGCACCAGAGCCAACACATCG |
| | hsa-miR-200b-3p | RT_200b-3p | #21 | GTTGGCTCTGGTGCAGGGTCCGAGGTATTCGCACCAGAGCCAACTCATCA |
| | hsa-miR-200c-3p | RT_200c-3p | #21 | GTTGGCTCTGGTGCAGGGTCCGAGGTATTCGCACCAGAGCCAACTCCATC |
| | hsa-miR-129-3p | RT_129-3p | #21 | GTTGGCTCTGGTGCAGGGTCCGAGGTATTCGCACCAGAGCCAACATACTT |
| | hsa-miR-431-5p | RT_431-5p | #21 | GTTGGCTCTGGTGCAGGGTCCGAGGTATTCGCACCAGAGCCAACTGCATG |
| | hsa-miR-127-5p | RT_127-5p | #21 | GTTGGCTCTGGTGCAGGGTCCGAGGTATTCGCACCAGAGCCAACATCAGA |
| | hsa-miR-433 | RT_433 | #21 | GTTGGCTCTGGTGCAGGGTCCGAGGTATTCGCACCAGAGCCAACACACCG |
| | hsa-miR-483-3p | RT_483-3p | #21 | GTTGGCTCTGGTGCAGGGTCCGAGGTATTCGCACCAGAGCCAACAAGACG |
| | RNU6B | RT_U6B | #21 | GTTGGCTCTGGTGCAGGGTCCGAGGTATTCGCACCAGAGCCAAAAAATATGG |
| **3'UTR site direct mutagenesis** | NOTCH1 | UTR-NOTCH-mut34F | - | ACACAGAACAGTCAGGTTTTTATTTATATGTACTGTTTTATCTG |
| | NOTCH1 | UTR-NOTCH-mut34R | - | CAGTACATATAAATAAAAACCTGACTGTTCTGTGTAAAATAAAAGTAC |
| | NOTCH1 | UTR-NOTCH-mut200F | - | CTTTGTTTCAGGTCCTGTATGTAGTTGTTCGTTG |
| | NOTCH1 | UTR-NOTCH-mut200R | - | ACAACTACATACAGGACCTGCTGAAACAAAGATTC |

***Universal Probe Library (Roche Diagnostics)**

**Supplementary Table 6 -** List of miRNA mimics from Applied Biosystems-Ambion used in transfection experiments**.**

| microRNA | Accession number | miRBase Stem-Loop Accession | miRBase Mature miRNA Accession |
|---|---|---|---|
| hsa-miR-34b-5p | PM10743 | MI0000742 | MIMAT0000685 |
| hsa-miR-34c-5p | PM11039 | MI0000743 | MIMAT0000686 |
| hsa-miR-200a-3p | PM10991 | MI0000737 | MIMAT0000682 |
| hsa-miR-200b-3p | PM10492 | MI0000342 | MIMAT0000318 |
| hsa-miR-200c-3p | PM11714 | MI0000650 | MIMAT0000617 |
| NC2 | AM17111 | | |

**Supplementary Table 7 - Genes showing highest concordance for tumor-associated CNVs among the tested paraganglioma samples (104 genes with p<0.01).** Gains are in red background, losses in green background**.** Hyperlinks to gene, chromosomal position, and, where appropriate, protein, are provided.

| | Gene | Official full name | Chromosome | Losses | Gains | p-value |
|---|---|---|---|---|---|---|
| 1 | IDUA | iduronidasi, alfa-L | 4p16.3 | 0 | 15 | 0.000002 |
| 2 | ARHGEF16 | Rho guanine nucleotide exchange factor (GEF) 16 | 1p36.3 | 0 | 13 | 0.000026 |
| 3 | FAM173A | family with sequence similarity 173, member A | 16p13.3 | 0 | 13 | 0.000026 |
| 4 | FBXL16 | F-box and leucine-rich repeat protein 16 | 16p13.3 | 0 | 13 | 0.000026 |
| 5 | MEGF6 | multiple EGF-like-domain 6 | 1p36.3 | 0 | 13 | 0.000026 |
| 6 | NOTCH1 | Neurogenic notch homolog protein 1 | 9q34.3 | 0 | 13 | 0.000026 |
| 7 | PRDM16 | PR domain containing 16 | 1p36.23-p33 | 0 | 13 | 0.000026 |
| 8 | ABCA2 | ATP-binding cassette, sub-family A (ABC1), member 2 | 9q34 | 0 | 12 | 0.000078 |
| 9 | C9orf142 | chromosome 9 open reading frame 142 | 9q34.3 | 0 | 12 | 0.000078 |
| 10 | CLIC3 | chloride intracellular channel 3 | 9q34.3 | 0 | 12 | 0.000078 |
| 11 | COL20A1 | collagen, type XX, alpha 1 | 20q13.33 | 0 | 12 | 0.000078 |
| 12 | JMJD8 | jumonji domain containing 8 | 16p13.3 | 0 | 12 | 0.000078 |
| 13 | METRN | meteorin, glial cell differentiation regulator | 16p13.3 | 0 | 12 | 0.000078 |
| 14 | MSLN | mesothelin | 16p13.3 | 0 | 12 | 0.000078 |
| 15 | PLCH2 | phospholipase C, eta 2 | 1p36.32 | 0 | 12 | 0.000078 |
| 16 | SSTR5 | somatostatin receptor 5 | 16p13.3 | 0 | 12 | 0.000078 |
| 17 | STUB1 | STIP1 homology and U-box containing protein 1, E3 ubiquitin protein ligase | 16p13.3 | 0 | 12 | 0.000078 |
| 18 | WDR24 | WD repeat domain 24 | 16p13.3 | 0 | 12 | 0.000078 |
| 19 | FAM20C | family with sequence similarity 20, member C | 7p22.3 | 2 | 15 | 0.000179 |
| 20 | ADAM8 | ADAM metallopeptidase domain 8 | 10q26.3 | 0 | 11 | 0.000221 |
| 21 | ADRA2C | adreno receptor alpha 2C | 4p16 | 0 | 11 | 0.000221 |
| 22 | ARFGAP1 | ADP-ribosylation factor GTPase activating protein 1 | 20q13.33 | 0 | 11 | 0.000221 |
| 23 | C1QTNF8 | C1q and tumor necrosis factor related protein 8 | 16p13.3 | 0 | 11 | 0.000221 |
| 24 | CACNA1H | calcium channel, voltage-dependent, T type, alpha 1H subunit | 16p13.3 | 0 | 11 | 0.000221 |
| 25 | CCDC78 | coiled-coil domain-containing protein 78 | 16p13.3 | 0 | 11 | 0.000221 |
| 26 | CHTF18 | CTF18, chromosome transmission fidelity factor 18 homolog (S. cerevisiae) | 16p13.3 | 0 | 11 | 0.000221 |
| 27 | HAGHL | hydroxyacylglutathione hydrolase-like | 16p13.3 | 0 | 11 | 0.000221 |
| 28 | LCNL1 | lipocalin-like 1 | 9q34.3 | 0 | 11 | 0.000221 |
| 29 | LOC146336 | SSTR5 antisense RNA 1 (non-protein coding) | 16p13.3 | 0 | 11 | 0.000221 |
| 30 | MSLNL | mesothelin-like | 16p13.3 | 0 | 11 | 0.000221 |
| 31 | NARFL | nuclear prelamin A recognition factor-like | 16p13.3 | 0 | 11 | 0.000221 |
| 32 | PANK4 | pantothenate kinase 4 | 1p36.32 | 0 | 11 | 0.000221 |
| 33 | RHBDL1 | rhomboid, veinlet-like 1 (Drosophila) | 16p13.3 | 0 | 11 | 0.000221 |
| 34 | RHOT2 | ras homolog gene family, member T2 | 16p13.3 | 0 | 11 | 0.000221 |

*Continued*

| | Gene | Official full name | Chromosome | Losses | Gains | p-value |
|---|---|---|---|---|---|---|
| 35 | RPUSD1 | RNA pseudouridylate synthase domain containing 1 | 16p13.3 | 0 | 11 | 0.000221 |
| 36 | TMEM41B | trans-membrane protein 41B | 11p15.4 | 11 | 0 | 0.000221 |
| 37 | FGFRL1 | fibroblast growth factor receptor-like 1 | 4p16 | 1 | 13 | 0.000257 |
| 38 | TUBGCP2 | tubulin, gamma complex associated protein 2 | 10q26.3 | 1 | 11 | 0.000257 |
| 39 | ATAD5 | ATPase family, AAA domain containing 5 | 17q11.2 | 10 | 0 | 0.000600 |
| 40 | C16orf13 | chromosome 16 open reading frame 13 | 16p13.3 | 0 | 10 | 0.000600 |
| 41 | FAM195A | family with sequence similarity 195, member A | 16p13.3 | 0 | 10 | 0.000600 |
| 42 | GNG13 | guanine nucleotide binding protein (G protein), gamma 13 | 16p13.3 | 0 | 10 | 0.000600 |
| 43 | PHACTR4 | phosphatase and actin regulator 4 | 1p35.3 | 10 | 0 | 0.000600 |
| 44 | WDR90 | WD repeat-containing protein 90 | 16p13.3 | 0 | 10 | 0.000600 |
| 45 | GALNS | galactosamine (N-acetyl)-6-sulfate sulfatase | 16q24.3 | 1 | 12 | 0.000699 |
| 46 | SAMD11 | sterile alpha motif domain containing 11 | 1p36.33 | 1 | 12 | 0.000699 |
| 47 | ADAP1 | ArfGAP with dual PH domains 1 | 7p22.3 | 0 | 9 | 0.001559 |
| 48 | C4orf42 | CTBP1 antisense RNA 1 (non-protein coding) | 4p16.3 | 0 | 9 | 0.001559 |
| 49 | CTBP1 | C-terminal binding protein 1 | 4p16 | 0 | 9 | 0.001559 |
| 50 | DVL1 | dishevelled, dsh homolog 1 (Drosophila) | 1p36 | 0 | 9 | 0.001559 |
| 51 | GET4 | golgi to ER traffic protein 4 homolog (S. cerevisiae) | 7p22.3 | 0 | 9 | 0.001559 |
| 52 | MIR4326 | microRNA 4326 | chr 20 | 0 | 9 | 0.001559 |
| 53 | MIR662 | microRNA 662 | 16p13.3 | 0 | 9 | 0.001559 |
| 54 | NMNAT1 | nicotinamide nucleotide adenylyl transferase 1 | 1p36.22 | 9 | 0 | 0.001559 |
| 55 | RBP7 | retinol binding protein 7, cellular | 1p36.22 | 9 | 0 | 0.001559 |
| 56 | SLC26A1 | solute carrier family 26 (sulfate transporter), member 1 | 4p16.3 | 0 | 9 | 0.001559 |
| 57 | SNN | stannin | 16p13 | 9 | 0 | 0.001559 |
| 58 | SYCE1 | synaptonemal complex central element protein 1 | 10q26.3 | 0 | 9 | 0.001559 |
| 59 | ZFPM1 | zinc finger protein, multitype 1 | 16q24.2 | 0 | 9 | 0.001559 |
| 60 | JAG2 | jagged 2 | 14q32 | 1 | 11 | 0.001797 |
| 61 | C19orf23 | CIRBP antisense RNA 1 (non-protein coding) | 19p13.3 | 2 | 12 | 0.003351 |
| 62 | C19orf24 | chromosome 19 open reading frame 24 | 19p13.3 | 2 | 12 | 0.003351 |
| 63 | CIRBP | cold inducible RNA binding protein | 19p13.3 | 2 | 12 | 0.003351 |
| 64 | MIDN | midnolin | 19p13.3 | 2 | 12 | 0.003351 |
| 65 | CPSF3L | cleavage and polyadenylation specific factor 3-like | 1p36.33 | 0 | 8 | 0.003898 |
| 66 | EAPP | E2F-associated phosphoprotein | 14q13.1 | 8 | 0 | 0.003898 |
| 67 | EFNA2 | ephrin-A2 | 19p13.3 | 0 | 8 | 0.003898 |
| 68 | GLTPD1 | glycolipid transfer protein domain containing 1 | 1p36.33 | 0 | 8 | 0.003898 |
| 69 | HES5 | hairy and enhancer of split 5 (Drosophila) | 1p36.32 | 0 | 8 | 0.003898 |
| 70 | RXRA | retinoid X receptor, alpha | 9q34.3 | 0 | 8 | 0.003898 |
| 71 | TAS1R3 | taste receptor, type 1, member 3 | 1p36.33 | 0 | 8 | 0.003898 |

*Supplementary Table 7 continued*

| | Gene | Official full name | Chromosome | Losses | Gains | p-value |
|---|---|---|---|---|---|---|
| 72 | TPPP | tubulin polymerization promoting protein | 5p15.3 | 0 | 8 | 0.003898 |
| 73 | WDR47 | WD repeat domain 47 | 1p13.3 | 8 | 0 | 0.003898 |
| 74 | ACAP3 | ArfGAP with coiled-coil, ankyrin repeat and PH domains 3 | chr 1 | 1 | 10 | 0.004387 |
| 75 | PUSL1 | pseudouridylate synthase-like 1 | 1p36.33 | 1 | 10 | 0.004387 |
| 76 | RHPN1 | rhophilin, Rho GTPase binding protein 1 | 8q24.3 | 1 | 10 | 0.004387 |
| 77 | SCNN1D | sodium channel, non-voltage-gated1, delta subunit | 1p36.3-36.2 | 1 | 10 | 0.004387 |
| 78 | NOC2L | nucleolar complex associated 2 homolog | 1p36.33 | 2 | 11 | 0.007841 |
| 79 | AKIRIN1 | akirin 1 | 1p34.3 | 7 | 0 | 0.009401 |
| 80 | BRD9 | bromo domain containing 9 | 5p15.33 | 0 | 7 | 0.009401 |
| 81 | C17orf42 | chromosome 17 open reading frame 42 | chr 17 | 7 | 0 | 0.009401 |
| 82 | C9orf139 | chromosome 9 open reading frame 139 | 9q34.3 | 0 | 7 | 0.009401 |
| 83 | CYP2E1 | cytochrome P450, family 2, subfamily E, polypeptide 1 | 10q24.3-qter | 0 | 7 | 0.009401 |
| 84 | DNLZ | DNL-type zinc finger protein | 9q34.3 | 0 | 7 | 0.009401 |
| 85 | DPP8 | dipeptidyl-peptidase 8 | 15q22 | 7 | 0 | 0.009401 |
| 86 | FUT7 | fucosyl transferase 7 (alpha (1,3) fucosyl transferase) | 9q34.3 | 0 | 7 | 0.009401 |
| 87 | GMEB1 | glucocorticoid modulatory element binding protein 1 | 1p35.3 | 7 | 0 | 0.009401 |
| 88 | GPSM1 | G-protein signaling modulator 1 | 9q34.3 | 0 | 7 | 0.009401 |
| 89 | IPP | intra-cisternal A particle-promoted polypeptide | 1p34-p32 | 7 | 0 | 0.009401 |
| 90 | KLHL17 | kelch-like 17 | 1p36.33 | 0 | 7 | 0.009401 |
| 91 | LRRC37A3 | leucine rich repeat containing 37, member A3 | 17q24.1 | 7 | 0 | 0.009401 |
| 92 | LZIC | leucine zipper and CTNNBIP1 domain containing | 1p36.22 | 7 | 0 | 0.009401 |
| 93 | PLEKHN1 | pleckstrin homology domain containing, family N member 1 | 1p36.33 | 0 | 7 | 0.009401 |
| 94 | RGS12 | regulator of G-protein signaling 12 | 4p16.3 | 0 | 7 | 0.009401 |
| 95 | RHBDL2 | rhomboid, veinlet-like 2 (Drosophila) | 1p34.3 | 7 | 0 | 0.009401 |
| 96 | SFPQ | splicing factor proline/glutamine-rich | 1p34.3 | 7 | 0 | 0.009401 |
| 97 | SLC6A3 | solute carrier family 6 (neurotransmitter transporter, dopamine), member 3 | 5p15.3 | 0 | 7 | 0.009401 |
| 98 | SPON2 | spondin 2, extracellular matrix protein | 4p16.3 | 0 | 7 | 0.009401 |
| 99 | SPRNP1 | shadow of prion protein homolog (zebrafish) pseudogene 1 | 10q26.3 | 0 | 7 | 0.009401 |
| 100 | UBE4B | ubiquitination factor E4B | 1p36.3 | 7 | 0 | 0.009401 |
| 101 | ZBTB8A | zinc finger and BTB domain containing 8A | 1p35.1 | 7 | 0 | 0.009401 |
| 102 | ZBTB8OS | zinc finger and BTB domain containing 8 opposite strand | 1p35.1 | 7 | 0 | 0.009401 |
| 103 | ZNF850 | zinc finger protein 850 | 19q13.12 | 7 | 0 | 0.009401 |
| 104 | ZYG11B | zyg-11 homolog B (C. elegans) | 1p32.3 | 7 | 0 | 0.009401 |

*Supplementary Table 7*

**Supplementary Table 8 - Most significant enriched biological themes and functional-related gene groups identified using DAVID (http://david.abcc.ncifcrf.gov) for the top 104 CNV-affected genes.** The top terms include “Notch signaling pathway”, “mesothelin”, “ArfGap”, “Rhomboid”, “GoLoco”. The table is ordered according to Bonferroni correction. Only the term “Notch signaling pathway” is statistically significant after Bonferroni and Benjamini corrections. Terms containing *NOTCH1* or molecules related to NOTCH1 signaling are in bold. Notably, 23 of the 48 partially-redundant terms include *NOTCH1* or genes interacting with *NOTCH1* (in bold).

| Category | Term | Count | Genes | P Value | Bonferroni | Benjamini |
|---|---|---|---|---|---|---|
| KEGG_PATHWAY | hsa04330:Notch signaling pathway | 5 | **CTBP1, NOTCH1, HES5, JAG2, DVL1** | 0,00006 | 0,002 | 0,002 |
| PIR_SUPERFAMILY | PIRSF030203:mesothelin | 2 | MSLN, MSLNL | 0,00917 | 0,333 | 0,333 |
| SMART | SM00105:ArfGap | 3 | ARFGAP1, ACAP3, ADAP1 | 0,00766 | 0,340 | 0,340 |
| PIR_SUPERFAMILY | PIRSF037470:rhomboid protein | 2 | RHBDL1, RHBDL2 | 0,01373 | 0,456 | 0,262 |
| PIR_SUPERFAMILY | PIRSF037470:Rhomboid | 2 | RHBDL1, RHBDL2 | 0,01373 | 0,456 | 0,262 |
| SMART | SM00390:GoLoco | 2 | RGS12, GPSM1 | 0,03270 | 0,834 | 0,592 |
| GOTERM_MF_FAT | GO:0008060~ARF GTPase activator activity | 3 | ARFGAP1, ACAP3, ADAP1 | 0,00838 | 0,838 | 0,838 |
| INTERPRO | IPR001164:Arf GTPase activating protein | 3 | ARFGAP1, ACAP3, ADAP1 | 0,00892 | 0,860 | 0,860 |
| INTERPRO | IPR010335:Pre-pro-megakaryocyte potentiating factor precursor | 2 | MSLN, MSLNL | 0,01018 | 0,894 | 0,674 |
| SMART | SM00233:PH | 5 | ACAP3, PLCH2, ARHGEF16, PLEKHN1, ADAP1 | 0,04124 | 0,897 | 0,531 |
| SMART | SM00504:Ubox | 2 | UBE4B, STUB1 | 0,04185 | 0,901 | 0,438 |
| SP_PIR_KEYWORDS | alternative splicing | 50 | **JAG2**, FAM195A, GET4, ZYG11B, DPP8, BRD9, GMEB1, ARHGEF16, WDR90, WDR47, ACAP3, C16ORF13, HAGHL, CPSF3L, ARFGAP1, RHPN1, PUSL1, ABCA2, PRDM16, STUB1, ZBTB8OS, RGS12, PLCH2, MSLN, GPSM1, SCNN1D, PHACTR4, C17ORF42, SAMD11, UBE4B, SYCE1, ATAD5, TUBGCP2, **DVL1**, C9ORF142, SFPQ, JMJD8, NARFL, RHOT2, RHBDL1, CHTF18, CACNA1H, WDR24, RHBDL2, PLEKHN1, MEGF6, TMEM41B, CCDC78, COL20A1, ZBTB8A | 0,01661 | 0,925 | 0,925 |
| GOTERM_MF_FAT | GO:0030695~GTPase regulator activity | 7 | ARFGAP1, RGS12, ACAP3, RHPN1, ARHGEF16, GPSM1, ADAP1 | 0,01239 | 0,932 | 0,740 |
| GOTERM_MF_FAT | GO:0060589~nucleoside-triphosphatase regulator activity | 7 | ARFGAP1, RGS12, ACAP3, RHPN1, ARHGEF16, GPSM1, ADAP1 | 0,01369 | 0,949 | 0,629 |
| INTERPRO | IPR017213:Peptidase S54, rhomboid, metazoan | 2 | RHBDL1, RHBDL2 | 0,01523 | 0,965 | 0,674 |
| UP_SEQ_FEATURE | domain:Arf-GAP | 3 | ARFGAP1, ACAP3, ADAP1 | 0,00902 | 0,976 | 0,976 |
| GOTERM_MF_FAT | GO:0005096~GTPase activator activity | 5 | ARFGAP1, RGS12, ACAP3, GPSM1, ADAP1 | 0,02082 | 0,989 | 0,679 |
| GOTERM_BP_FAT | GO:0032312~regulation of ARF GTPase activity | 3 | ARFGAP1, ACAP3, ADAP1 | 0,00636 | 0,990 | 0,990 |
| GOTERM_MF_FAT | GO:0034450~ubiquitin-ubiquitin ligase activity | 2 | UBE4B, STUB1 | 0,02365 | 0,994 | 0,644 |
| UP_SEQ_FEATURE | domain:EGF-like 5; calcium-binding | 3 | **NOTCH1, JAG2, MEGF6** | 0,01542 | 0,998 | 0,959 |
| INTERPRO | IPR003109:GoLoco motif | 2 | RGS12, GPSM1 | 0,03518 | 1,000 | 0,859 |
| GOTERM_BP_FAT | GO:0030182~neuron differentiation | 7 | **NOTCH1, HES5, EFNA2, RXRA, JAG2, SPON2, DVL1** | 0,01088 | 1,000 | 0,981 |
| GOTERM_BP_FAT | GO:0048667~cell morphogenesis involved in neuron differentiation | 5 | **NOTCH1, EFNA2, RXRA, SPON2, DVL1** | 0,01226 | 1,000 | 0,950 |
| GOTERM_MF_FAT | GO:0005083~small GTPase regulator activity | 5 | ARFGAP1, ACAP3, RHPN1, ARHGEF16, ADAP1 | 0,04172 | 1,000 | 0,784 |
| GOTERM_BP_FAT | GO:0048812~neuron projection morphogenesis | 5 | **NOTCH1, EFNA2, RXRA, SPON2, DVL1** | 0,01307 | 1,000 | 0,909 |
| INTERPRO | IPR002610:Peptidase S54, rhomboid | 2 | RHBDL1, RHBDL2 | 0,04501 | 1,000 | 0,867 |
| INTERPRO | IPR003613:U box domain | 2 | UBE4B, STUB1 | 0,04501 | 1,000 | 0,867 |
| GOTERM_BP_FAT | GO:0048663~neuron fate commitment | 3 | **NOTCH1, HES5, JAG2** | 0,01394 | 1,000 | 0,870 |
| UP_SEQ_FEATURE | domain:EGF-like 26 | 2 | **NOTCH1, MEGF6** | 0,02538 | 1,000 | 0,971 |
| UP_SEQ_FEATURE | domain:EGF-like 20 | 2 | **NOTCH1, MEGF6** | 0,02538 | 1,000 | 0,971 |
| UP_SEQ_FEATURE | domain:EGF-like 24 | 2 | **NOTCH1, MEGF6** | 0,02538 | 1,000 | 0,971 |
| UP_SEQ_FEATURE | domain:EGF-like 27 | 2 | **NOTCH1, MEGF6** | 0,02538 | 1,000 | 0,971 |
| GOTERM_BP_FAT | GO:0050909~sensory perception of taste | 3 | GNG13, TAS1R3, SCNN1D | 0,01523 | 1,000 | 0,845 |
| GOTERM_BP_FAT | GO:0032012~regulation of ARF protein signal transduction | 3 | ARFGAP1, ACAP3, ADAP1 | 0,01523 | 1,000 | 0,845 |
| UP_SEQ_FEATURE | splice variant | 49 | **JAG2**, FAM195A, GET4, ZYG11B, DPP8, BRD9, GMEB1, ARHGEF16, WDR90, WDR47, ACAP3, C16ORF13, HAGHL, CPSF3L, ARFGAP1, RHPN1, PUSL1, ABCA2, PRDM16, STUB1, ZBTB8OS, RGS12, PLCH2, MSLN, GPSM1, SCNN1D, C17ORF42, PHACTR4, SAMD11, UBE4B, SYCE1, ATAD5, **DVL1**, C9ORF142, SFPQ, JMJD8, NARFL, RHOT2, RHBDL1, CHTF18, CACNA1H, WDR24, RHBDL2, PLEKHN1, MEGF6, TMEM41B, CCDC78, COL20A1, ZBTB8A | 0,02825 | 1,000 | 0,948 |
| GOTERM_BP_FAT | GO:0060120~inner ear receptor cell fate commitment | 2 | **HES5, JAG2** | 0,01704 | 1,000 | 0,833 |
| GOTERM_BP_FAT | GO:0009912~auditory receptor cell fate commitment | 2 | **HES5, JAG2** | 0,01704 | 1,000 | 0,833 |
| UP_SEQ_FEATURE | domain:EGF-like 22 | 2 | **NOTCH1, MEGF6** | 0,03038 | 1,000 | 0,921 |
| GOTERM_BP_FAT | GO:0000902~cell morphogenesis | 6 | **NOTCH1, EFNA2, RXRA, SPON2, DVL1, IDUA** | 0,01799 | 1,000 | 0,808 |
| UP_SEQ_FEATURE | domain:U-box | 2 | UBE4B, STUB1 | 0,03535 | 1,000 | 0,916 |
| UP_SEQ_FEATURE | domain:EGF-like 4 | 3 | **NOTCH1, JAG2, MEGF6** | 0,03572 | 1,000 | 0,882 |
| GOTERM_BP_FAT | GO:0000904~cell morphogenesis involved in differentiation | 5 | **NOTCH1, EFNA2, RXRA, SPON2, DVL1** | 0,02049 | 1,000 | 0,813 |
| GOTERM_BP_FAT | GO:0048858~cell projection morphogenesis | 5 | **NOTCH1, EFNA2, RXRA, SPON2, DVL1** | 0,02076 | 1,000 | 0,783 |
| GOTERM_BP_FAT | GO:0032990~cell part morphogenesis | 5 | **NOTCH1, EFNA2, RXRA, SPON2, DVL1** | 0,02394 | 1,000 | 0,799 |
| GOTERM_BP_FAT | GO:0031175~neuron projection development | 5 | **NOTCH1, EFNA2, RXRA, SPON2, DVL1** | 0,02394 | 1,000 | 0,799 |
| UP_SEQ_FEATURE | zinc finger region:C4-type | 3 | ARFGAP1, ACAP3, ADAP1 | 0,04764 | 1,000 | 0,919 |
| GOTERM_BP_FAT | GO:0032989~cellular component morphogenesis | 6 | **NOTCH1, EFNA2, RXRA, SPON2, DVL1, IDUA** | 0,02728 | 1,000 | 0,813 |
| GOTERM_BP_FAT | GO:0007409~axonogenesis | 4 | **NOTCH1, EFNA2, RXRA, SPON2** | 0,04981 | 1,000 | 0,943 |

**Supplementary Table 9 – Fractions of mutation carriers and of cases with tumor-associated loss of SDHB in the subsets of paragangliomas analyzed for germline *SDH* mutations.** Germline *SDH* gene mutation analyses, performed in 34 cases for *SDHB*, *SDHD*, and *SDHC*, and in 9 cases for *SDHAF2*, identified 13 mutation carriers (38.2%), of which 5/34 (14.7%) in *SDHB*; 1/34 (2.9%) in *SDHC*; 6/34 (17.6%) in *SDHD*; 1/9 (11.1%) in *SDHAF2*. Furthermore, CNV and gene-centric analyses identified a large germline SDHB deletion in one additional case (17.1% of *SDHB* mutation carriers; 40% of total mutation carriers).

| | Mutation | Fraction with SDHB loss | | Age (range, mean) | Gender |
|---|---|---|---|---|---|
| ***SDHB*** | 6/35 (17.1%) | 6/6 (100%) | | 25-55, 35,3 | 3F, 3M |
| ***SDHC*** | 1/34 (2,9%) | 0/1 (0%) | | 43 | 1M |
| ***SDHD*** | 6/34 (17.6%) | 5/6 (83,3%) | | 31-59, 47,5 | 4F, 2M |
| ***SDHAF2*** | 1/9 (11.1%) | 1/1 (100%) | | 24 | 1M |
| **Total Mutated** | 14/35 (40%) | 12/14 (85,7%) | $P$=0,0019 | 24-59, 45,6 | 7F, 7M |
| **Undetected** | 21/35 (60%) | 6/20 (30%) | | 25-67, 45,8 | 14F, 7M |

**Supplementary Table 10 - Individual characteristics, *SDH* mutations, and loss of SDHB immunostaining in paraganglioma subsets with different clinical presentation.** SDHB loss was detected 9/11 multiple/recurrent paraganglioma (81.8%) and was less frequent in the tympano-jugular (9/28, 32.1%) and tympanic (1/4, 25%) tumors. The unique metastatic tumor (34PTJ) and the single vagal PGL (PV-06/R18) resulted SDHB-negative.

| Presentation | Age (range, mean) (46 cases) | Gender (46 cases) | Fraction with *SDH* mutations (35 cases)* | Fraction with SDHB loss (45 cases)[#] |
|---|---|---|---|---|
| **multiple/recurrent (11)** | 25-59, 43.7 | 6F, 5M | 7/10 (70%) | 9/11 (81.8%) |
| **metastatic** | 25 | 1F | 1/1 (100%) | 1/1 (100%) |
| **tympano-jugular (29)** | 24-67, 45.8 | 18F, 11M | 5/23 (21.7%) | 9/28 (32.1%) |
| **tympanic (4)** | 58-73, 58.7 | 3F, 1M | na | 1/4 (25%) |
| **vagal (1)** | 27 | 1F | 1/1 (100%) | 1/1(100%) |
| **total cases (46)** | 24-73, 45.6 | 29F, 17M | 14/35 (40%) | 21/45 (46.6%) |

*Cases with known mutation status in *SDH* genes;
[#]SDHB immunohistochemistry not assessable for one case due to poor sample quality.

**Supplementary Table 11 -** Mean NOTCH1 and JAG2 immunostaining intensities (± SD) in chief cells (CC), sustentacular cells (SC) and endothelial cells (EC) of paraganglioma subsets defined according to *SDH* mutation status (detected, undetected) and to SDHB immunohistochemistry (IHC, negative or positive). Endothelial cells are negative for JAG2.

| | NOTCH1 IHC intensity (± SD) | | | *P* | JAG2 IHC intensity (± SD) | | *P* |
|---|---|---|---|---|---|---|---|
| | **CC** | **SC** | **EC** | | **CC** | **SC** | |
| ***SDH* mutation** | | | | | | | |
| **detected (14)** | 1.76 (0.43) | 1.90 (0.43) | 2.76 (0.43) | ns | 1.00 (0.32) | 1.20 (0.52) | ns |
| **undetected (21)** | 1.78 (0.42) | 1.92 (0.61) | 2.78 (0.42) | | 0.78 (0.42) | 0.92 (0.61) | |
| **SDHB IHC** | | | | | | | |
| **negative (21)** | 1.76 (0.44) | 1.95 (0.50) | 2.76 (0.44) | ns | 0.9 (0.44) | 1.15 (0.74) | ns |
| **positive (24)** | 1.91 (0.51) | 2.04 (0.64) | 2.78 (0.42) | | 1.00 (0.31) | 1.23 (0.53) | |
