# Supplementary material for: Integrative genetic, epigenetic and pathological analysis of paraganglioma reveals complex dysregulation of NOTCH signaling
Source: Acta Neuropathol. 2013 Aug 18;126(4):575–94. doi: 10.1007/s00401-013-1165-y (PMC3789891; doi:10.1007/s00401-013-1165-y)
Supplement: Supplementary file 2 — Supplementary material (PDF 7790 kb) [file 401_2013_1165_MOESM2_ESM.pdf]

Online Resource 2

INTEGRATIVE GENETIC, EPIGENETIC AND PATHOLOGICAL ANALYSIS OF PARAGANGLIOMA REVEALS COMPLEX DYSREGULATION OF NOTCH SIGNALING

Alessandro Cama, Fabio Verginelli, Lavinia Vittoria Lotti Francesco Napolitano, Annalisa Morgano, Andria D'Orazio, Michele Vacca, Silvia Perconti, Felice Pepe, Federico Romani, Francesca Vitullo, Filippo di Lella, Rosa Visone, Massimo Mannelli, Hartmut P.H. Neumann, Giancarlo Raiconi, Carlo Paties, Antonio Moschetta, Roberto Tagliaferri, Angelo Veronese, Mario Sanna, Renato Mariani-Costantini.

Corresponding author: Professor Renato Mariani-Costantini, MD, Unit of General Pathology, Aging Research Center (Ce.S.I.), *G. d'Annunzio* University Foundation, *Via Colle dell'Ara,* 66100 Chieti, Italy. Tel. +39-0871541496, e-mail: rmc@unich.it

The file contains Supplementary Figures showing: Jacobson's nerve morphology (**Supplementary Fig. 1**); histoarchitecture of paraganglioma (**Supplementary Fig. 2**); single-channel grayscale images for the merged colocalizations of CTBP1 and S100 (**Supplementary Fig. 3**); qRT-PCR validation for representative miRNAs upregulated or downregulated in paragangliomas (**Supplementary Fig. 4**); list of miRNA families targeting the 3'UTR of NOTCH1 by TargetScan prediction algorithm (**Supplementary Fig. 5**); characterization of the expression of the miR-34b-5p, miR-34c-5p and miR-200b-3p in the PTJ64 tumor, derived PTJ64p cells, and PTJ64p cells infected with lentiviruses transducing miRs **(Supplementary Fig. 6).**

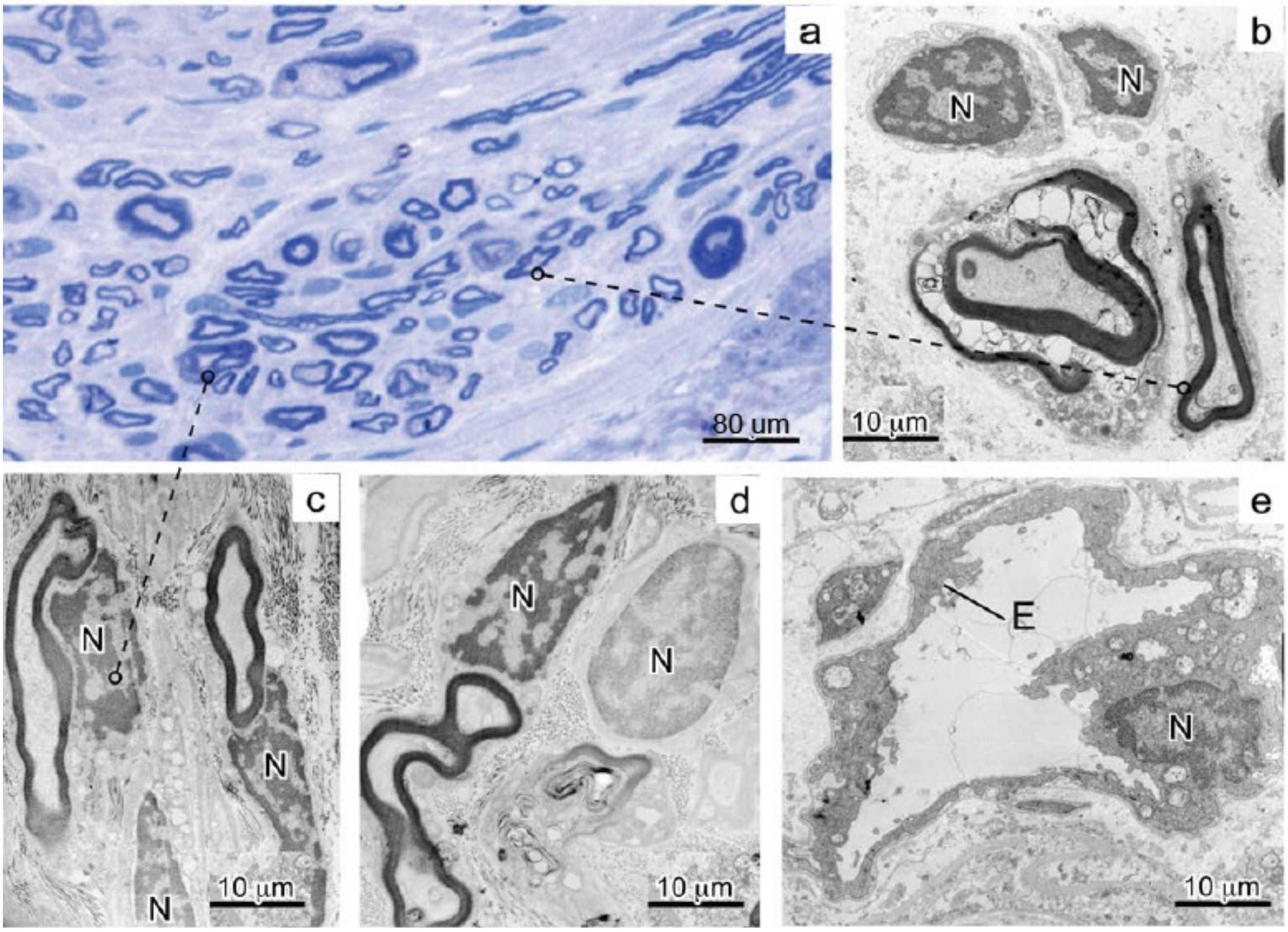

Supplementary Fig. 1 - Morphology of the Normal Jacobson's nerve samples. Panel a shows a toluidine-blue-stained semithin section of Jacobson's nerve with fascicles of morphologically normal myelinated axons, as seen by conventional light microscopy. Panels b, c, and d show electron micrographs of the same nerve, highlighting normal Schwann (glial) cells adjacent to myelinated axons. Panel e provides an ultrastructural view of a nerve capillary with plump endothelium. N = nucleus; E = endothelium, scale bars: a = 80 μm; b-e = 10 μm.

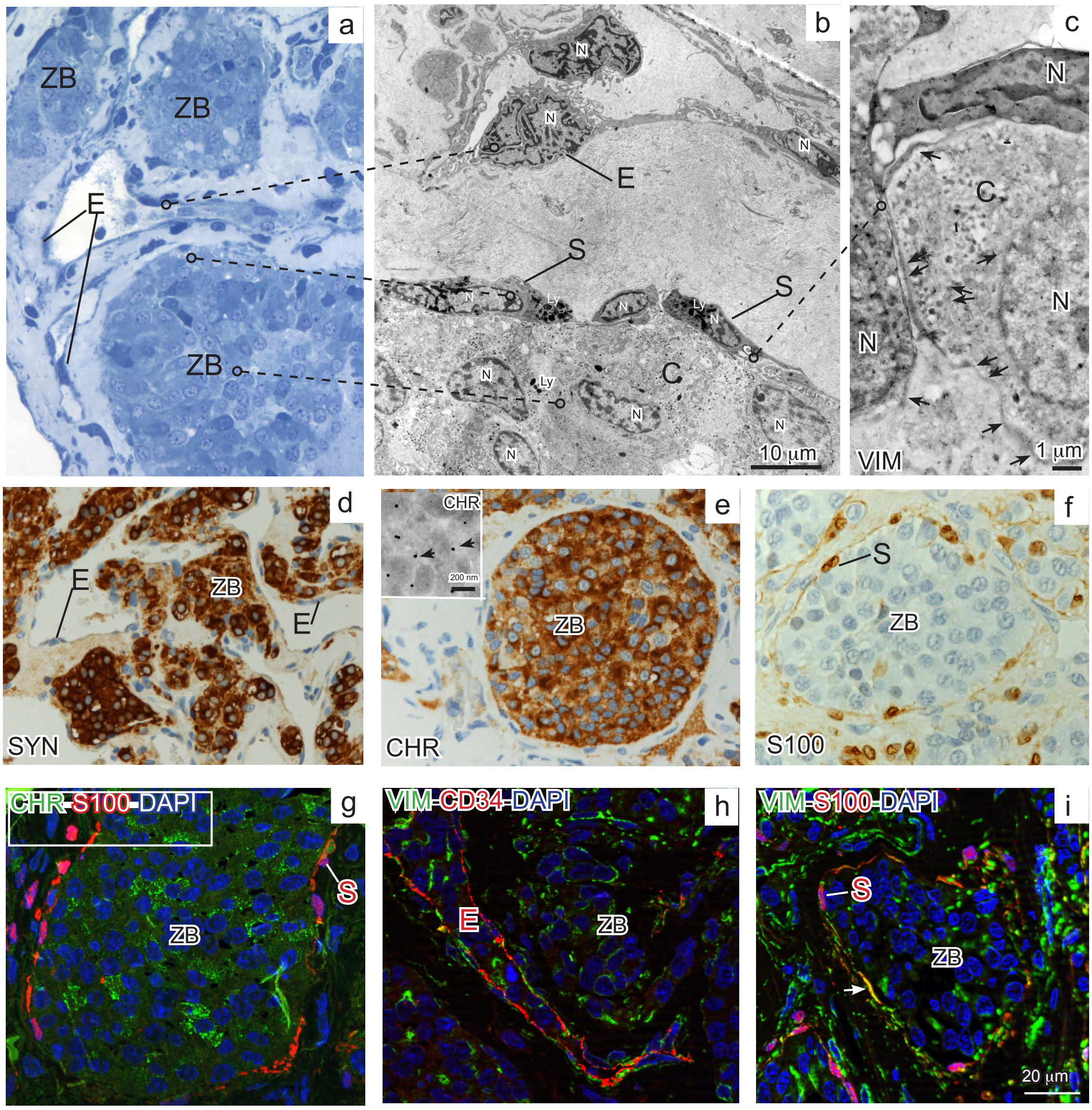

Supplementary Fig. 2 - Histoarchitecture of paraganglioma. Panels a, b and c show the basic structure and principal cell types of a typical paraganglioma, observed respectively by light microscopy on toluidine blue-stained semi-thin section (a), high resolution transmission electron microscopy (b) and cryo-immunoelectronmicroscopy (c). Panels d-i show the immunophenotypic characterization of the tumor cell types, as seen by immunoperoxidase staining on paraffin-embedded sections (d-f) and by Apotome immunofluorescence on DAPI-counterstained cryosections (g-i). The tumor consists in cohesive cell nests ("zellballens"), embedded in a stroma containing capillaries lined by plump endothelium (b) with pleomorphic nuclei and microvilli-like plasma membrane projections (b, arrow). The zellballen cells, strongly positive for the synaptic vesicle glycoprotein synaptophysin (d), include two distinct phenotypes, chief (neurosecretory) and sustentacular (glial) cells (b, c). The chief cells, that predominate, are round to polygonal elements characterized by neurosecretory granules (b, c, e1) positive for chromogranin A (e, g, e1). The sustentacular cells, spindle-shaped and generally less numerous, concentrate at the zellballen's periphery (a, b). These cells are strongly labeled with the glial marker S100 protein (f, g, i) and characteristically show thin, threadlike S100- and vimentin-labeled processes that extend around and within the zellballen (c, f, g, i). Ultrastructurally, the sustentacular cells show elongated nuclei and often contain heterogeneous cytoplasmic electron dense material, morphologically consistent with lysosomes, residual bodies or lipofuscin (b). The endothelia stain positive for the hematopoietic and endothelial progenitor cell marker CD34 (h) and for the mesenchymal marker vimentin (h, i). However, vimentin filaments are also present in chief and sustentacular cells (c, h, i). C = chief cell; CHR = chromogranin A; E = endothelium; Ly = lysosomes; N = nucleus; S = sustentacular cell; SG = secretory granules; SYN = synaptophysin; VIM = vimentin; ZB = zellballen. Bars: panels a, d, e, f, g, h, i = 20 μm; b = 10 μm; c = 1 μm; inset e1 = 200 nm.

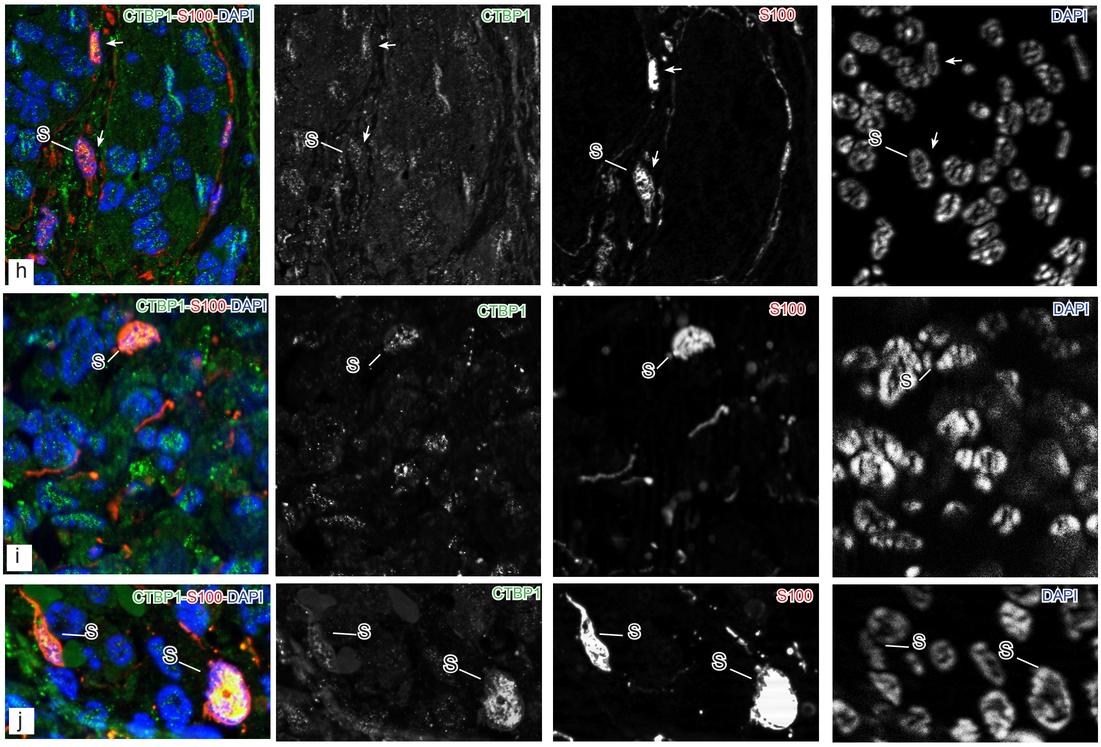

Supplementary Fig. 3 - Original single-channel grayscale images for the merged colocalizations of CTBP1 and S100 shown in Figure 3, panels h, i and j.

a

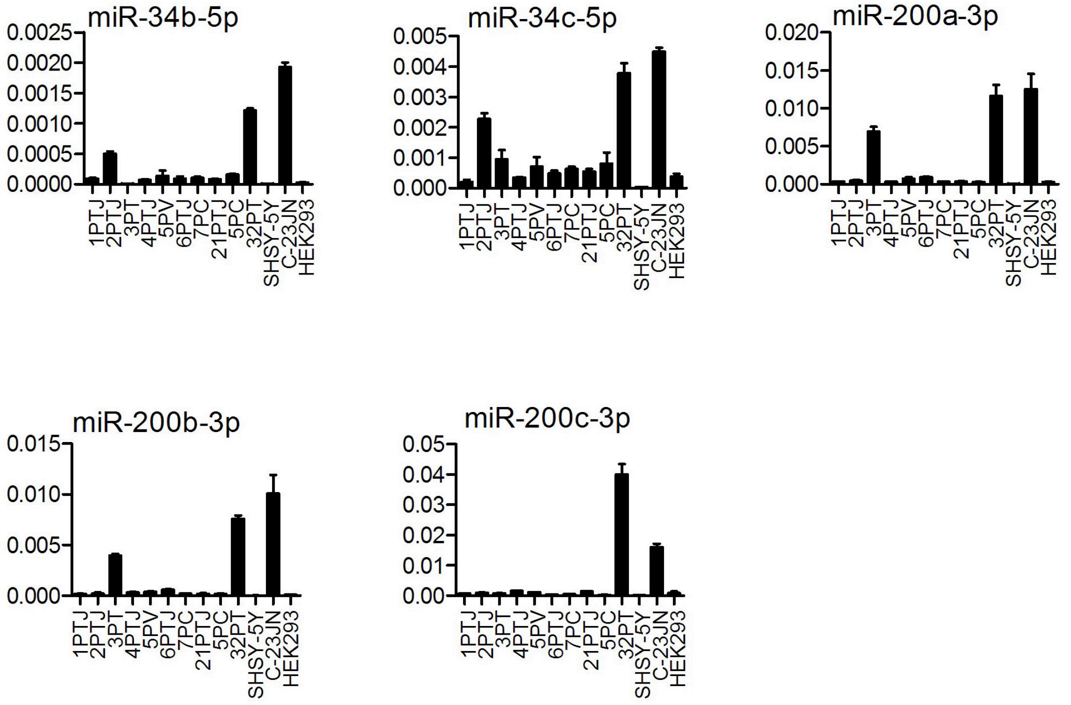

b

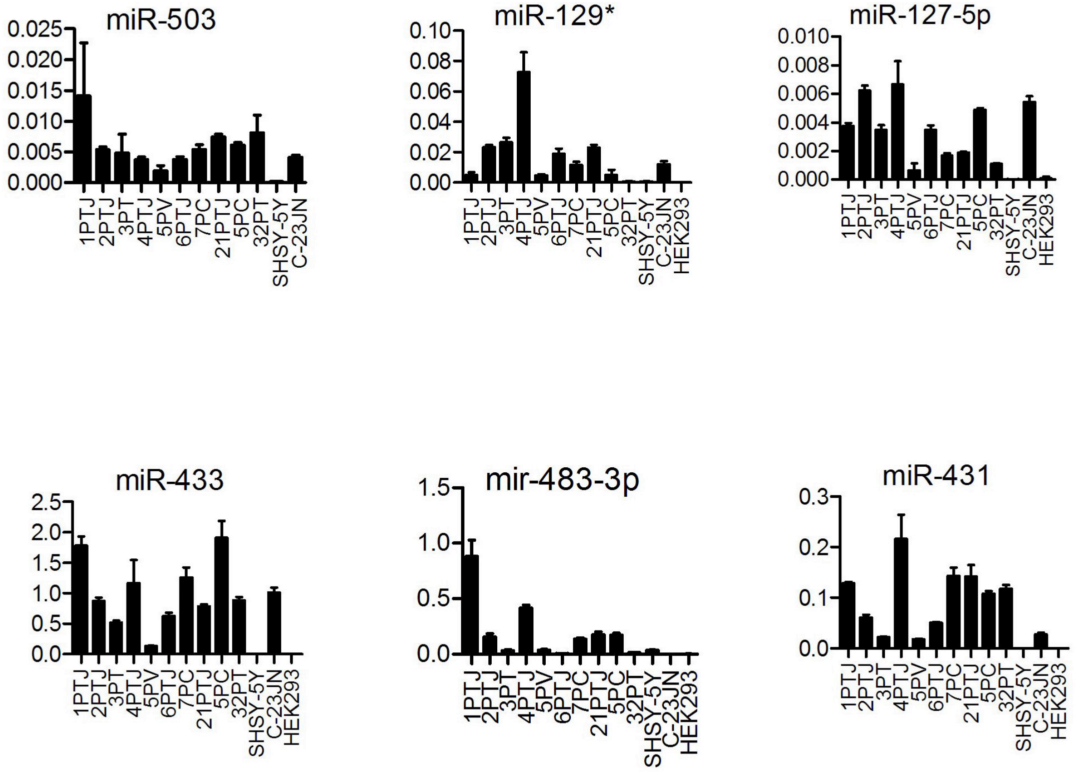

Supplementary Fig. 4 - Quantitative RT-PCR for representative microRNAs upregulated or downregulated in paragangliomas. Representative microRNAs downregulated (miR-34b-5p, miR-34c-5p, miR-200a, miR-200b and miR-200c) (panel a) or upregulated (miR-503, miR-129*, miR-431, miR-127-5p, miR-433 and miR-483-3p) (panel b) were analyzed by qRT-PCR on total RNA from 10 PGL samples, Jacobson's nerve (C-23JN) and the cell lines SH-SY5Y and HEK293.

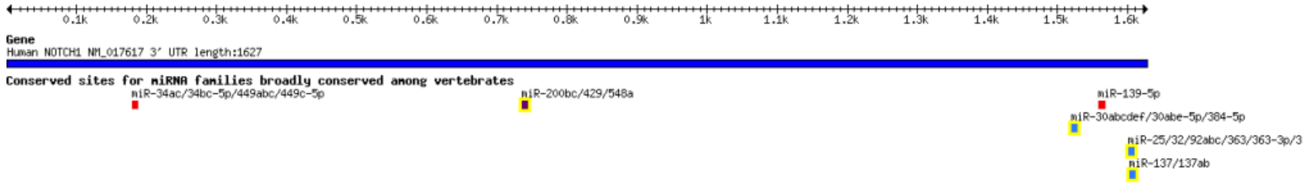

**miRNA families broadly conserved among vertebrates targeting 3'UTR NOTCH1 gene**
**miR-34ac/34bc-5p/449abc/449c-5p**
miR-139-5p
miR-25/32/92abc/363/363-3p/367
miR-137/137ab
**miR-200bc/429/548a (miR-8)**
miR-30abcdef/30abe-5p/384-5p

miR-34 family (miR-34ac/34bc-5p/449abc/449c-5p)

predicted consequential pairing of target region (top) and miRNA (bottom)

Position 180-186 of NOTCH1 3' UTR

hsa-miR-449a

```
5' ...UAUUUUACACAGAAACACUGCCU...
                     |||||||
3'    UGGUCGAUUGUUAUGUGACGGU
```

Position 180-186 of NOTCH1 3' UTR

hsa-miR-449b

```
5' ...UAUUUUACACAGAAACACUGCCU...
                     |||||||
3'    CGGUCGAUUGUUAUGUGACGGA
```

Position 180-186 of NOTCH1 3' UTR

hsa-miR-34a

```
5' ...UAUUUUACACAGAAACACUGCCU...
                     |||||||
3'    UGUUGGUCGAUUCUGUGACGGU
```

Position 180-186 of NOTCH1 3' UTR

hsa-miR-34c-5p

```
5' ...UAUUUUACACAGAAACACUGCCU...
                     |||||||
3'   CGUUAGUCGAUUGAUGUGACGGA
```

miR-8 family (miR-200bc/429/548a)

predicted consequential pairing of target region (top) and miRNA (bottom)

Position 736-743 of NOTCH1 3' UTR

hsa-miR-429

```
5' ...UCUUUGUUUCAGGUUCAGUAUUA...
                     |||||||
3'    UGCCAAAAUGGUCUGUCAUAAU
```

Position 736-743 of NOTCH1 3' UTR

hsa-miR-200c

```
5' ...UCUUUGUUUCAGGUUCAGUAUUA...
                     |||||||
3'   AGGUAGUAAUGGGCCGUCAUAAU
```

Position 736-743 of NOTCH1 3' UTR

hsa-miR-200b

```
5' ...UCUUUGUUUCAGGUUCAGUAUUA...
                     |||||||
3'    AGUAGUAAUGGUCCGUCAUAAU
```

Supplementary Fig. 5 - miRNA families broadly conserved among vertebrates targeting the 3'UTR of the *NOTCH1* gene by TargetScan prediction algorithm. Schematic representations of ~1.6 Kb of the 3'UTR and of the 6 conserved microRNA families predicted to target *NOTCH1* are indicated. In bold the miR-34ac/34bc-5p/449abc/449c-5p and miR-200bc/429/548a (miR-8) families and their target nucleotide sequences in the 3'UTR of *NOTCH1*.

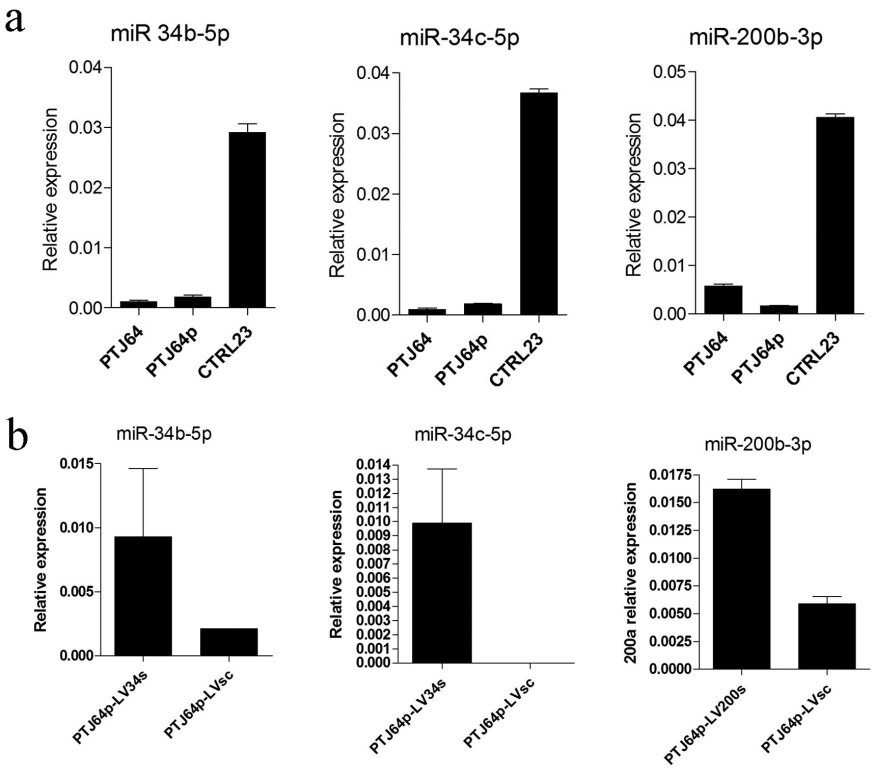

Supplementary Fig. 6 – Characterization of the expression of miR-34b-5p, miR-34c-5p and miR-200b-3p in PTJ64 tumor, derived PTJ64p cells, and PTJ64p cells infected with lentiviruses transducing miRs. Panel a: by qRT-PCR, both the original tympano-jugular paraganglioma sample PTJ64 and the derived primary paraganglioma cell culture PTJ64p show downregulation of miR-34b-5p, miR-34c-5p and miR-200b-3p, compared to the histogenetically relevant tissue control CTRL23 (Jacobson's nerve). Panel b: infection with lentiviral vectors transducing the miR-34s and miR-200s clusters (LV34s, LV200s) results in significant ectopic expression of miR-34b-5p, miR-34c-5p, and miR-200b-3p in primary paraganglioma PTJ64p cells. Error bars represent standard deviation.
