# Supplementary material for: Integrative genetic, epigenetic and pathological analysis of paraganglioma reveals complex dysregulation of NOTCH signaling
Source: Acta Neuropathol. 2013 Aug 18;126(4):575–94. doi: 10.1007/s00401-013-1165-y (PMC3789891; doi:10.1007/s00401-013-1165-y)
Supplement: Supplementary file 3 — Supplementary material (PDF 29 kb) [file 401_2013_1165_MOESM3_ESM.pdf]

Online Resource 3

INTEGRATIVE GENETIC, EPIGENETIC AND PATHOLOGICAL ANALYSIS OF PARAGANGLIOMA REVEALS COMPLEX DYSREGULATION OF NOTCH SIGNALING

Alessandro Cama, Fabio Verginelli, Lavinia Vittoria Lotti Francesco Napolitano, Annalisa Morgano, Andria D'Orazio, Michele Vacca, Silvia Perconti, Felice Pepe, Federico Romani, Francesca Vitullo, Filippo di Lella, Rosa Visone, Massimo Mannelli, Hartmut P.H. Neumann, Giancarlo Raiconi, Carlo Paties, Antonio Moschetta, Roberto Tagliaferri, Angelo Veronese, Mario Sanna, Renato Mariani-Costantini.

Corresponding author: Professor Renato Mariani-Costantini, MD, Unit of General Pathology, Aging Research Center (Ce.S.I.), *G. d'Annunzio* University Foundation, *Via Colle dell'Ara*, 66100 Chieti, Italy. Tel. +39-0871541496, e-mail: rmc@unich.it.

Orthogonal validation of CNV hits:

Appendix 1 - Orthogonal validation of CNV hits at *NOTCH1*, *JAG2*, *HES5*, *DVL1* and *CTBP1*.
Appendix 2 - Orthogonal validation of CNV hits at *AKIRIN1*, *IDUA*, *PHACTR4* and *SDHB*.

Appendix 1 - Orthogonal validation of CNV hits at *NOTCH1*, *JAG2*, *HES5*, *DVL1* and *CTBP1*.

Samples	NOTCH1					JAG2					HES5					DVL1					CTBP1				
	PennCNV	NFMP				PennCNV	QPCR				PennCNV	QPCR				PennCNV	NFMP				PennCNV	QPCR			
		Predicted	Calculated	SD	CV%		Predicted	Calculated	SD	CV%		Predicted	Calculated	SD	CV%		Predicted	Calculated	SD	CV%		Predicted	Calculated	SD	CV%
1B	no change	no change	0,95	0,09	9,15	no change	no change	2,14	0,12	5,77	no change	no change	2,30	0,21	9,09	no change	no change	0,99	0,09	9,47	no change	no change	1,94	0,21	10,98
1PTJ	gain	gain	1,50	0,10	6,39	gain	no change	1,93	0,10	5,43	no change	gain	3,52	0,18	5,15	gain	gain	1,31	0,12	8,88	no change	no change	2,39	0,23	9,75
2B	no change	no change	0,95	0,00	0,36	no change	no change	1,87	0,13	6,82	no change	no change	1,50	0,18	12,09	no change	no change	0,94	0,10	11,14	no change	no change	1,84	0,20	11,14
2PTJ	no change	no change	1,22	0,13	11,03	no change	no change	1,85	0,13	6,78	no change	no change	1,66	0,19	11,64	no change	n.a.	n.a.	n.a.	n.a.	no change	no change	2,11	0,22	10,22
3B	no change	no change	1,25	0,11	8,82	no change	no change	2,16	0,13	5,91	no change	n.a.	n.a.	n.a.	n.a.	no change	no change	1,21	0,10	8,17	no change	no change	2,40	0,22	9,12
3PT	gain	no change	1,20	0,12	9,95	gain	no change	2,00	0,10	4,94	no change	no change	1,84	0,21	11,17	gain	n.a.	n.a.	n.a.	n.a.	gain	gain	2,53	0,22	8,86
4B	no change	no change	0,99	0,06	6,46	no change	no change	2,09	0,12	5,96	no change	no change	1,97	0,18	9,25	no change	n.a.	n.a.	n.a.	n.a.	no change	no change	2,15	0,21	9,56
4PTJ	gain	gain	1,60	0,16	10,16	gain	gain	3,01	0,14	4,52	gain	gain	4,35	0,20	4,49	no change	gain	1,57	0,18	11,72	gain	gain	4,24	0,21	4,99
5B	no change	no change	1,13	0,02	1,47	no change	no change	2,19	0,14	6,35	no change	gain	2,78	0,19	6,71	no change	no change	1,08	0,06	5,90	no change	gain	3,00	0,21	6,90
5PV	gain	n.a.	n.a.	n.a.	n.a.	no change	no change	1,67	0,12	7,08	no change	gain	3,14	0,20	6,52	gain	n.a.	n.a.	n.a.	n.a.	gain	gain	2,57	0,24	9,44
5PC	gain	gain	1,48	0,25	17,23	gain	gain	3,22	0,13	3,99	no change	gain	3,66	0,18	4,92	no change	n.a.	n.a.	n.a.	n.a.	no change	gain	2,76	0,23	8,27
6B	no change	no change	0,94	0,06	6,77	no change	no change	1,97	0,10	5,22	no change	no change	2,04	0,18	8,88	no change	no change	0,98	0,15	15,50	no change	no change	2,37	0,21	8,99
6PTJ	gain	no change	1,20	0,16	13,43	gain	gain	3,53	0,13	3,67	gain	gain	3,14	0,20	6,38	gain	no change	0,84	0,05	6,31	gain	gain	3,63	0,22	5,93
7B	no change	no change	1,00	0,05	5,50	no change	no change	2,09	0,10	4,90	no change	no change	1,54	0,19	12,46	no change	gain	1,34	0,07	5,09	no change	no change	1,75	0,23	13,14
7PC	gain	no change	1,05	0,09	8,70	gain	no change	2,27	0,10	4,38	gain	gain	3,33	0,19	5,71	gain	gain	1,61	0,20	12,36	no change	no change	2,04	0,22	10,73
8B	gain	no change	1,07	0,16	14,52	gain	no change	1,87	0,11	5,73	no change	no change	1,84	0,19	10,33	no change	no change	1,20	0,09	7,28	no change	no change	2,33	0,21	8,97
8PTJ	gain	no change	1,09	0,18	16,87	no change	no change	2,03	0,12	5,93	no change	no change	1,87	0,22	11,64	no change	n.a.	n.a.	n.a.	n.a.	gain	gain	4,50	0,23	5,05
11B	gain	no change	0,95	0,13	13,30	no change	no change	1,83	0,10	5,40	no change	loss	1,35	0,18	13,33	no change	no change	0,84	0,08	9,03	no change	gain	2,54	0,23	9,06
11PT	gain	no change	0,86	0,08	9,74	no change	no change	1,94	0,13	6,60	no change	no change	1,50	0,19	12,81	no change	no change	0,96	0,08	8,58	gain	gain	2,94	0,23	7,98
12B	no change	no change	1,24	0,14	10,94	no change	no change	2,38	0,11	4,65	no change	no change	2,32	0,18	7,79	no change	no change	1,20	0,11	9,26	no change	no change	2,14	0,22	10,24
12PTJ	no change	no change	1,11	0,17	15,65	no change	no change	1,61	0,12	7,77	no change	loss	1,40	0,20	14,28	no change	no change	1,09	0,07	6,81	no change	no change	1,60	0,23	14,29
13B	no change	n.a.	n.a.	n.a.	n.a.	no change	no change	1,86	0,10	5,52	no change	no change	2,26	0,21	9,29	no change	n.a.	n.a.	n.a.	n.a.	no change	no change	2,00	0,24	11,78
13PTJ	gain	gain	1,34	0,18	12,12	no change	no change	2,08	0,10	4,68	no change	no change	1,91	0,23	12,18	no change	no change	0,85	0,02	2,07	no change	gain	2,72	0,21	7,86
14B	no change	n.a.	n.a.	n.a.	n.a.	no change	no change	1,93	0,14	7,07	no change	no change	2,23	0,22	9,67	no change	no change	1,21	0,17	14,31	no change	no change	1,95	0,20	10,51
14PT	no change	no change	1,22	0,09	7,72	no change	no change	1,92	0,12	6,02	no change	gain	2,56	0,21	8,38	no change	n.a.	n.a.	n.a.	n.a.	no change	no change	1,82	0,20	11,22
19B	no change	n.a.	n.a.	n.a.	n.a.	no change	n.a.	n.a.	n.a.	n.a.	no change	n.a.	n.a.	n.a.	n.a.	no change	n.a.	n.a.	n.a.	n.a.	no change	no change	2,35	0,27	11,45
19PT	no change	n.a.	n.a.	n.a.	n.a.	no change	no change	1,94	0,10	5,05	no change	no change	1,72	0,19	11,15	no change	n.a.	n.a.	n.a.	n.a.	no change	no change	2,06	0,21	10,06
20B	no change	no change	0,94	0,05	5,26	no change	no change	2,07	0,10	4,83	no change	n.a.	n.a.	n.a.	n.a.	no change	n.a.	n.a.	n.a.	n.a.	no change	no change	1,82	0,23	12,46
20PT	no change	no change	0,88	0,06	6,74	gain	no change	1,89	0,15	8,06	gain	no change	1,95	0,18	9,39	gain	n.a.	n.a.	n.a.	n.a.	gain	no change	2,29	0,22	9,69
21B	no change	no change	0,90	0,09	9,86	no change	no change	1,99	0,13	6,33	no change	no change	2,20	0,19	8,64	no change	no change	0,99	0,13	13,59	no change	no change	1,87	0,21	11,08
21PTJ	gain	gain	1,31	0,15	11,24	gain	no change	2,36	0,10	4,19	gain	no change	2,02	0,20	9,80	no change	no change	1,02	0,10	9,73	gain	gain	3,35	0,22	6,48
32B	no change	no change	0,95	0,05	4,75	no change	no change	1,73	0,10	5,99	no change	no change	2,11	0,19	9,13	no change	n.a.	n.a.	n.a.	n.a.	no change	gain	3,10	0,21	6,73
32PT	gain	gain	1,69	0,16	9,21	gain	gain	2,96	0,14	4,57	gain	gain	3,38	0,19	5,70	gain	no change	1,19	0,11	9,00	gain	gain	2,98	0,21	6,90
33B	no change	no change	1,11	0,01	0,56	no change	no change	1,87	0,13	7,11	no change	no change	1,65	0,18	11,01	no change	n.a.	n.a.	n.a.	n.a.	no change	no change	1,93	0,21	10,86
33PT-1	no change	no change	1,03	0,10	9,56	no change	no change	1,92	0,11	5,58	no change	no change	1,77	0,18	10,22	no change	n.a.	n.a.	n.a.	n.a.	no change	no change	1,53	0,23	14,80
34B	no change	no change	1,09	0,01	1,34	gain	no change	2,07	0,10	4,92	no change	n.a.	n.a.	n.a.	n.a.	no change	no change	0,98	0,11	11,22	gain	no change	1,91	0,21	10,86
34PTJ	gain	no change	0,91	0,06	6,85	gain	no change	2,03	0,10	4,85	gain	loss	1,38	0,20	14,15	gain	n.a.	n.a.	n.a.	n.a.	gain	no change	1,68	0,23	13,91
36B	no change	no change	1,15	0,08	6,80	no change	no change	2,02	0,11	5,33	no change	gain	3,34	0,18	5,39	no change	no change	1,15	0,10	8,72	no change	gain	3,78	0,20	5,41
36PT	gain	gain	1,67	0,25	14,81	gain	gain	2,54	0,10	3,94	gain	gain	4,20	0,18	4,31	no change	n.a.	n.a.	n.a.	n.a.	no change	gain	3,72	0,34	9,04
37B	no change	no change	0,98	0,07	7,35	no change	no change	1,71	0,12	6,89	no change	no change	2,09	0,18	8,85	no change	no change	0,81	0,13	15,57	no change	no change	1,93	0,22	11,33
37PTJ	gain	gain	2,13	0,17	8,18	gain	gain	2,85	0,11	3,83	no change	gain	8,60	0,18	2,14	gain	gain	2,73	0,13	4,60	no change	no change	2,23	0,20	9,17
43B	gain	n.a.	n.a.	n.a.	n.a.	no change	no change	1,83	0,10	5,68	no change	no change	1,64	0,18	11,04	no change	n.a.	n.a.	n.a.	n.a.	no change	no change	1,83	0,21	11,26
43PTJ	gain	gain	1,68	0,09	5,27	no change	no change	2,24	0,11	5,05	no change	gain	3,79	0,19	4,94	no change	no change	1,29	0,23	17,40	no change	gain	3,13	0,23	7,35
44B	no change	no change	1,02	n.a.	15,98	no change	no change	2,15	0,10	4,54	no change	no change	1,98	0,18	9,18	no change	n.a.	n.a.	n.a.	n.a.	no change	no change	1,88	0,22	11,65
44PT	no change	n.a.	n.a.	n.a.	n.a.	no change	no change	2,17	0,15	7,02	no change	loss	1,36	0,19	13,97	no change	n.a.	n.a.	n.a.	n.a.	no change	no change	1,56	0,24	15,61
45B	no change	no change	1,09	0,16	14,83	no change	no change	2,12	0,11	4,96	no change	no change	1,61	0,21	13,04	no change	n.a.	n.a.	n.a.	n.a.	no change	no change	1,61	0,21	12,98
45PTJ	gain	no change	1,10	0,09	7,80	no change	no change	1,97	0,11	5,67	no change	no change	2,18	0,21	9,53	no change	no change	1,02	0,14	14,20	no change	no change	2,31	0,21	8,90

Appendix 2 - Orthogonal validation of CNV hits at *AKIRIN1*, *IDUA*, *PHACTR4* and *SDHB*.

Samples	AKIRIN1					IDUA					PHACTR4					SDHB				
	PennCNV	QPCR				PennCNV	QPCR				PennCNV	QPCR				PennCNV	NFMP			
		Predicted	Calculated	SD	CV%		Predicted	Calculated	SD	CV%		Predicted	Calculated	SD	CV%		Predicted	Calculated	SD	CV%
1B	no change	no change	1,98	0,12	5,94	no change	no change	1,63	0,17	10,41	no change	no change	1,88	0,15	7,99	no change	no change	0,99	0,08	7,68
1PTJ	no change	no change	1,56	0,11	6,81	no change	no change	1,72	0,16	9,18	no change	loss	1,44	0,14	9,70	no change	no change	0,93	0,05	4,95
2B	no change	no change	1,97	0,12	5,88	no change	no change	1,99	0,18	8,81	no change	no change	1,89	0,13	6,86	no change	no change	0,96	0,07	6,81
2PTJ	no change	no change	1,70	0,16	9,12	gain	no change	2,33	0,17	7,13	loss	no change	1,49	0,14	9,51	no change	no change	0,88	0,01	1,15
3B	no change	no change	1,89	0,12	6,58	gain	no change	2,24	0,16	7,07	no change	no change	2,32	0,14	6,12	no change	no change	0,97	0,03	2,72
3PT	no change	no change	1,52	0,14	8,93	gain	no change	1,95	0,16	8,03	no change	no change	1,66	0,14	8,46	no change	no change	0,86	0,02	2,08
4B	no change	no change	2,05	0,12	5,66	no change	no change	1,84	0,16	8,53	no change	no change	1,86	0,15	8,24	no change	no change	1,04	0,04	4,07
4PTJ	loss	loss	1,19	0,11	9,01	gain	gain	2,77	0,16	5,68	loss	loss	1,30	0,13	10,23	no change	loss	0,66	0,01	2,04
5B	no change	no change	2,23	0,12	5,26	no change	no change	2,33	0,18	7,75	no change	no change	2,05	0,13	6,20	no change	no change	1,13	0,07	6,59
5PV	no change	no change	1,80	0,17	9,20	no change	no change	1,69	0,18	10,42	no change	no change	1,96	0,13	6,87	no change	no change	0,89	0,05	5,60
5PC	no change	no change	2,02	0,12	6,11	gain	no change	2,38	0,17	7,16	no change	no change	1,91	0,14	7,25	no change	no change	0,98	0,01	1,35
6B	no change	no change	1,85	0,12	6,67	no change	no change	2,16	0,16	7,35	no change	no change	2,02	0,14	6,80	no change	no change	1,01	0,03	3,00
6PTJ	no change	no change	1,91	0,12	6,18	gain	gain	2,55	0,15	5,97	no change	no change	1,88	0,18	9,76	no change	no change	0,73	0,02	2,93
7B	no change	no change	1,89	0,12	6,41	no change	no change	1,81	0,16	8,77	no change	no change	2,09	0,13	6,36	no change	no change	1,06	0,06	5,60
7PC	no change	no change	1,87	0,15	7,79	gain	gain	2,64	0,18	6,77	no change	no change	1,63	0,13	8,06	no change	no change	0,92	0,05	5,26
8B	no change	no change	2,21	0,12	5,60	no change	no change	2,10	0,16	7,55	no change	no change	1,96	0,13	6,59	loss	loss	0,51	0,02	3,09
8PTJ	no change	no change	1,86	0,12	6,60	no change	no change	2,07	0,17	8,32	no change	no change	1,66	0,16	9,93	loss	loss	0,49	0,02	4,91
11B	no change	no change	2,07	0,12	5,86	no change	no change	2,05	0,16	7,82	no change	no change	2,28	0,14	6,22	no change	no change	0,95	0,02	2,60
11PT	no change	no change	1,97	0,11	5,46	gain	gain	2,51	0,30	11,97	no change	no change	2,10	0,13	6,21	no change	no change	0,93	0,03	3,28
12B	no change	no change	2,04	0,12	5,74	no change	gain	2,63	0,15	5,82	no change	no change	1,95	0,15	7,71	no change	no change	1,03	0,01	1,30
12PTJ	no change	no change	1,69	0,11	6,32	gain	no change	1,85	0,16	8,79	no change	no change	2,07	0,15	7,11	no change	no change	0,98	0,02	2,55
13B	no change	no change	1,91	0,14	7,39	no change	no change	1,72	0,16	9,11	no change	no change	2,27	0,15	6,50	no change	no change	1,06	0,04	3,66
13PTJ	loss	loss	1,13	0,11	9,83	gain	no change	2,24	0,15	6,80	loss	loss	1,09	0,13	11,47	loss	loss	0,63	0,00	0,50
14B	no change	no change	1,94	0,11	5,47	no change	no change	2,08	0,17	7,99	no change	no change	1,92	0,14	7,01	no change	no change	1,05	0,02	1,63
14PT	no change	no change	1,82	0,12	6,36	no change	no change	2,11	0,18	8,57	no change	no change	2,03	0,13	6,18	no change	no change	0,90	0,05	5,87
19B	no change	no change	2,19	0,15	6,88	no change	no change	2,29	0,21	9,12	no change	no change	2,28	0,13	5,53	no change	na	na	na	na
19PT	no change	no change	1,83	0,12	6,63	no change	gain	2,66	0,16	5,87	no change	no change	2,00	0,13	6,45	no change	na	na	na	na
20B	no change	no change	1,88	0,11	5,72	no change	gain	2,67	0,18	6,64	no change	no change	2,04	0,13	6,13	no change	no change	1,00	0,02	2,06
20PT	no change	no change	1,61	0,11	7,10	gain	no change	2,40	0,18	7,39	loss	no change	2,30	0,14	6,30	no change	no change	0,90	0,03	3,45
21B	no change	no change	1,77	0,12	6,89	no change	no change	1,97	0,17	8,45	no change	no change	2,01	0,15	7,59	no change	no change	0,96	0,02	2,37
21PTJ	loss	loss	1,18	0,16	13,45	gain	no change	2,33	0,17	7,51	loss	loss	1,25	0,13	10,75	no change	no change	0,79	0,10	12,72
32B	no change	no change	1,97	0,12	5,95	no change	no change	2,04	0,15	7,47	no change	no change	2,15	0,13	5,97	no change	no change	0,96	0,00	0,17
32PT	loss	loss	0,89	0,12	13,85	gain	gain	2,84	0,17	5,94	loss	loss	0,98	0,13	13,17	no change	loss	0,65	0,02	3,72
33B	no change	no change	1,70	0,11	6,25	no change	no change	2,13	0,16	7,46	no change	no change	1,57	0,16	10,12	no change	no change	0,93	0,01	1,75
33PT-1	loss	loss	1,12	0,12	10,38	gain	no change	2,09	0,15	7,34	no change	no change	1,74	0,13	7,64	no change	no change	0,87	0,03	3,90
34B	no change	no change	2,08	0,14	6,66	gain	gain	2,64	0,17	6,48	no change	gain	2,79	0,13	4,71	no change	no change	0,99	0,10	9,61
34PTJ	loss	loss	1,31	0,12	9,06	gain	no change	2,37	0,17	7,36	no change	no change	1,56	0,13	8,66	no change	no change	0,82	0,05	6,43
36B	no change	no change	2,13	0,11	5,00	no change	no change	2,28	0,28	12,11	no change	no change	1,90	0,13	6,95	no change	no change	1,10	0,09	8,00
36PT	loss	loss	1,22	0,11	9,20	no change	no change	2,42	0,17	6,97	loss	no change	1,64	0,13	7,87	no change	no change	0,85	0,11	13,17
37B	no change	no change	1,92	0,11	5,54	no change	no change	1,93	0,17	8,68	no change	no change	1,67	0,15	9,20	no change	no change	0,94	0,03	2,92
37PTJ	no change	no change	1,64	0,11	6,69	gain	gain	3,74	0,19	5,18	loss	no change	1,66	0,13	7,58	no change	no change	0,98	0,05	5,08
43B	no change	no change	1,88	0,12	6,13	no change	no change	1,83	0,16	8,56	no change	no change	1,83	0,16	8,93	no change	no change	0,98	0,04	3,72
43PTJ	no change	no change	1,69	0,13	7,73	gain	gain	2,68	0,15	5,68	no change	no change	2,06	0,14	6,77	no change	no change	0,89	0,04	4,31
44B	no change	no change	2,05	0,12	5,62	no change	no change	1,77	0,16	8,99	no change	no change	1,90	0,14	7,27	no change	no change	0,99	0,00	0,27
44PT	no change	no change	1,88	0,13	6,84	gain	gain	2,83	0,31	11,08	loss	loss	1,39	0,15	11,04	no change	no change	1,00	0,01	1,18
45B	no change	no change	2,23	0,12	5,47	no change	no change	1,88	0,17	8,78	no change	no change	2,11	0,15	7,09	no change	no change	1,03	0,03	2,44
45PTJ	no change	no change	2,05	0,13	6,38	no change	no change	2,18	0,18	8,45	loss	no change	2,08	0,13	6,25	no change	no change	0,94	0,05	5,54
